# Incorporation of an Isohexide Subunit into the Endochin-like Quinolone Scaffold

**DOI:** 10.3390/molecules29153615

**Published:** 2024-07-31

**Authors:** Julia Senkina, Spencer Knapp

**Affiliations:** Department of Chemistry and Chemical Biology, Rutgers, The State University of New Jersey, 123 Bevier Road, Piscataway, NJ 08854, USA; julia.senkina@yale.edu

**Keywords:** malaria, isoidide, Suzuki coupling, endochin like quinolones, Mitsunobu reaction, cyt bc1 complex

## Abstract

In order to improve the drug-likeness qualities, the antimalarial endochin-like quinolone (ELQ) scaffold has been modified by replacing the 4-(trifluoromethoxy)phenyl portion with an isoidide unit that is further adjustable by varying the distal O-substituents. As expected, the water solubilities of the new analogs are greatly improved, and the melting points are lower. However, the antimalarial potency of the new analogs is reduced to EC_50_ > 1 millimolar, a result ascribable to the hydrophilic nature of the new substitution.

## 1. Introduction

**Figure 1:**
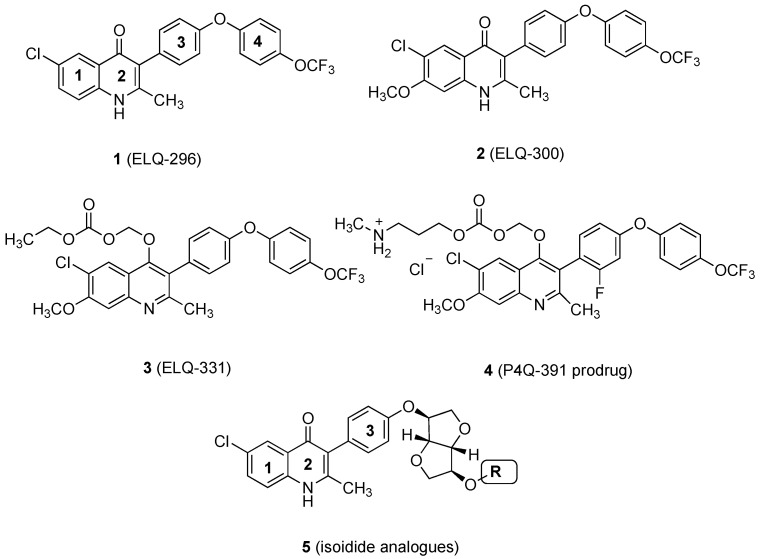
Structures of bioactive ELQs and a template (**5**) for isoidide incorporation.

Elaboration of the endochin-like quinolone (ELQ) scaffold has led to the development of several potent and target-selective antimalarials, including the 4-[4-(trifluoromethoxy)phenoxy]phenyl derivatives ELQ-296 and ELQ-300 (**1** and **2**, respectively, Figure 1) [1]. These compounds target the cytochrome bc1 complex, also known as Complex III, in the *Plasmodium* mitochondrial electron transport chain [2,3]. However, the drug-likeness qualities of the molecules fall short of what might be required of an effective malaria drug, which includes their poor water solubility, high crystallinity, and limited bioavailability [2,4].

Research efforts to improve the bioavailability of ELQs were largely centered on the preparation of prodrug derivatives of ELQs, such as the *O*-(ethoxycarboxymethyl derivative, **3** [5], and the methylaminopropoxycarboxymethyl derivative, **4** [6]. Such prodrugs have limitations, however, in that their cleavage characteristics may differ as a function of species, tissue distribution, and local enzymatic or pH environments [7,8,9,10,11]. Adjustment of the drug-likeness properties of the active compound itself offers the potential advantage that its behavior in biological systems is more easily defined and evaluated.

As part of our program to improve drug-likeness properties early in the discovery process for drug candidates, we have found that incorporation or attachment of an isohexide unit to the bioactive scaffold can improve the solubility, permeability, and metabolic stability [12]. In the best examples, this can be accomplished without sacrificing potency or safety from off-target effects [12]. Incorporation of an isohexide unit into the ELQ scaffold might be achieved by attachment at any of several positions, including the quinolone nitrogen (N-1) and the quinolone benzo ring (e.g., C-7), or by replacement of the trifluoromethoxyphenyl substituent (see **5**, Figure 1). The quinolone NH is required for efficient target binding [3], so it ought to remain unmodified. Attachment of a (polar) amino-adenine group at C-7 has recently been explored as a means to augment the effectiveness of quinolone antimalarials. Similarly, aza substitution (N for CH) in the central phenyl ring of the 3-aryl substituent (ring 3) has been investigated; the presence of one nitrogen is well tolerated, but double nitrogen substitution is deleterious [1]. There is little published information available on the effect of introducing additional oxygen-containing substituents on rings 3 or 4. However, we chose to incorporate the isoidide (both ring substituents *exo*) unit as a distal part of the 3-aryl substituent (see Figure 1, structure **5**) for three reasons: (1) we could maintain the optimized quinolone portion of the ELQ’s along with the favorable chloro substituent (compare **1**) [1,3,13,14], (2) the isoidide substructure could be attached by relatively straightforward and well-precedented Suzuki coupling methods [3,15], and (3) the remaining isoidide hydroxyl could be modified to adjust the size and polarity of the resulting analogs. It remained to be seen whether potency would be sacrificed as a result of these changes.

## 2. Results and Discussion

### 2.1. Preparation of the Suzuki Coupling Partners

**Scheme 1:**
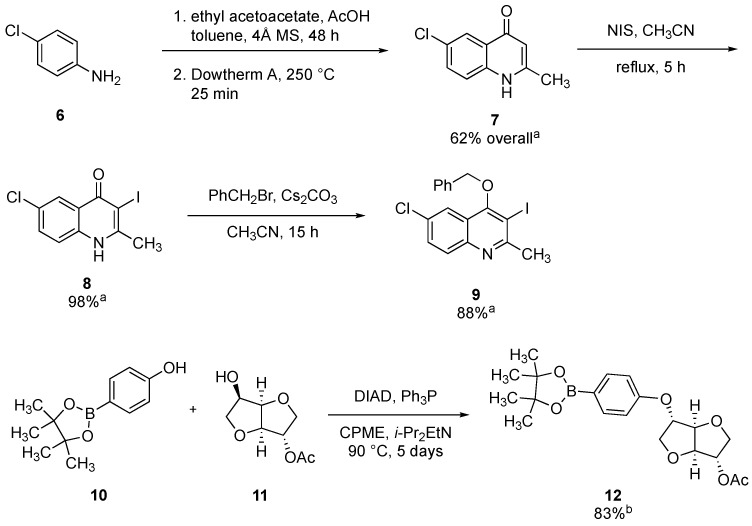
Synthesis of the Suzuki coupling partners **9** and **12** for isohexide incorporation into ELQs. (a) The product was purified by crystallization or precipitation; (b) the product was purified by chromatography.

The elaboration of 4-chloraniline (**6**, Scheme 1) into 3-iodoquinolone **8** followed the literature procedures [1,2,16] with minor modifications to improve the yields. For the Conrad–Limpach cyclization, nearly quantitative formation of the initially coupled enamine was seen when molecular sieves were used as the dehydrating reagent in toluene solution at room temperature. N-iodosuccinimide (NIS) was found to be a superior reagent for ring iodination [17]. The O-benzyl protecting group was chosen because it can be attached in high yield and offers several possibilities for deprotection under mild conditions. The alkylation product **9** precipitates directly from the reaction mixture. Previous ELQ syntheses used O-methyl or O-ethyl groups [1,2].

Commercial 4-hydroxyphenylboronate pinacol ester (**10**) was coupled to commercial 4-O-acetylisosorbide by a Mitsunobu reaction in a manner similar to reactions recently described for isosorbide itself [12]. The reaction is slow but efficient; the purification of **12** requires silica chromatography. Cyclopentyl methyl ether (CPME) proved to be superior to THF for the Mitsunobu coupling in that it is higher boiling, easier to keep dry, and peroxide-free [18]. 

### 2.2. Suzuki Coupling and Preparation of ELQ Analogues

**Scheme 2:**
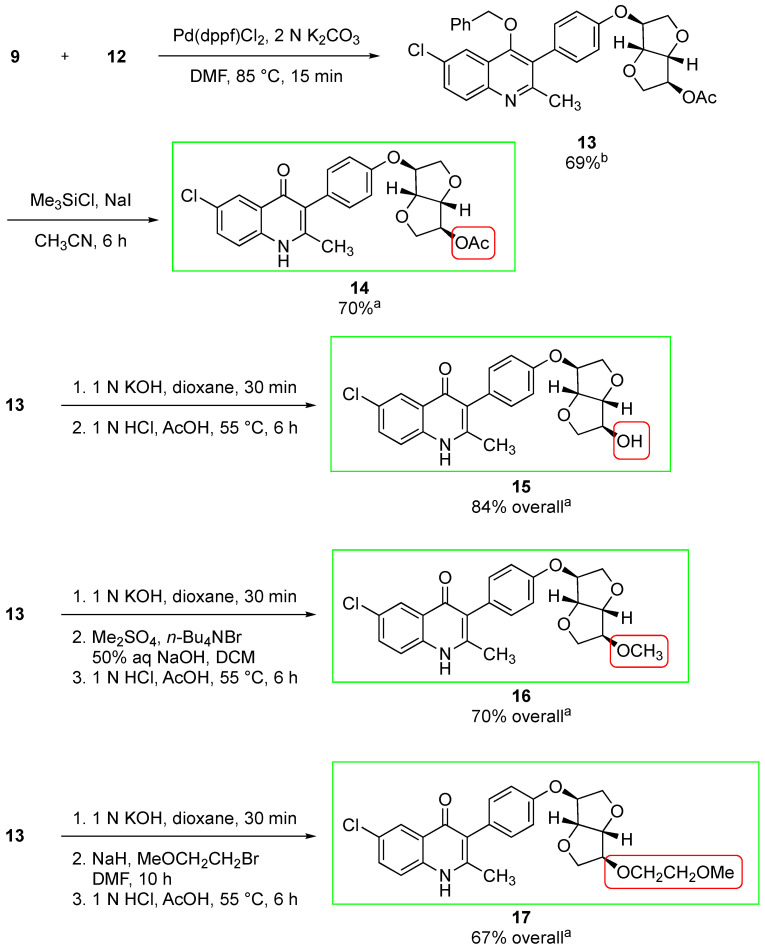
Suzuki coupling to form the ELQ framework and further modification of the terminal hydroxy substituent. O-substituents are shown in the red boxes, and the final four ELQ analogs are shown in the green boxes. (a) The product was purified by crystallization or precipitation; (b) the product was purified by chromatography.

The Suzuki coupling reaction was successful with Pd(dppf)Cl_2_ as the palladium source [3] (see **13**, Scheme 2), requiring only 15 min at 85 °C. Further heating caused partial base-promoted cleavage of the acetyl group. The O-benzyl protecting group was efficiently removed by nucleophilic de-alkylation to give the O-acetyl ELQ analog **14**, which precipitated directly from the reaction mixture. 

Purposeful deacetylation of the Suzuki product **13** was followed by the removal of the O-benzyl protecting group, this time with dilute HCl, to give the ELQ analog **15**. O-methylation of the deacylated compound from **13** followed by debenzylation as before provided the methoxy analog **16**, and O-alkylation with bromomethyl ethyl ether followed by debenzylation as before led to the O-methoxyethyl analog **17**. In Scheme 2, the distal isoidide substituents (–OAc, –OH, –OMe, and –OCH_2_CH_2_OMe, respectively) are indicated by red boxes, and the final four analogs (**14**–**17**) are shown in green boxes.

### 2.3. Evaluation of ELQ Analogues

Data from the physicochemical and in vitro evaluation of ELQs **1** and **2**, along with the new ELQ analogs **14**–**17**, are displayed in Table 1. Replacement of the terminal 4-(trifluoromethoxy)phenyl group by the substituted isoidide unit is conducted with very little change in molecular weight (second column). However, the incorporation of the isoidide substructure leads to a dramatic decrease in ClogP, roughly three log units (third column). This is the expected result of the introduction of several new oxygen functionalities and the replacement of the lipophilic aryl ring. There is also a modest but definite reduction in melting point when going from the simple ELQs to the isoidide analogs (fourth column). The non-planar bicyclic ether rings may well reduce pi stacking in the crystal lattice, and a reduced melting point is often predictive of improved solubility [19]. Likewise, the isoidide analogs **15**, **16**, and **17** show much improved measured kinetic solubility in aqueous buffer over ELQ-300 (sixth column), as expected. None of the new analogs is cytotoxic to human BJ fibroblasts below 100 micromolar (seventh column).

Evaluation of the new analogs **14**–**17** against the chloroquine-sensitive *P. falciparum* strain 3D7 (fifth column) indicates that potency below 1 millimolar has been lost as a result of the structural changes to the ELQ scaffold. This disappointing result invalidates our initial assumption that these structural changes far from the quinolone ring could be imposed as a means to improve the drug-like qualities. Evidently, the introduction of polar oxygen functionality in place of the lipophilic 4-(trifluoromethoxy)phenyl subunit is detrimental to binding at the presumed Plasmodium cytochrome bc1 target. Docking studies conducted by Capper et al. [21] on ELQ-structurally-related 4(1H)-pyridones with bovine cyt bc1 suggest that the diphenyl ether substituent extends towards the entrance of the cavity between cytochrome bc1 monomers. While the end of the putative binding channel features several hydrophobic residues, the protein environment next to heme b_h_ is somewhat polar due to the presence of His201, Ser35, and Asp228 residues. Whether new groups introduced into the ELQ scaffold near or beyond the trifluoromethoxy group would be substantially solvent-exposed, a desirable situation for isohexide incorporation to be non-detrimental to target binding, has not been established.

## 3. Materials and Methods

### 3.1. General

All commercially available solvents, reagents, and compounds were used as received. Gravity chromatography was performed by using silica gel (Sorbent Technologies 230–400 mesh) as the stationary phase. Silica gel 60 F_254_ pre-coated plates were used for thin-layer chromatography, and visualization was accomplished with UV light (254 nm) or ninhydrin stain. The LC–MS and high-resolution mass spectrometry (HRMS) analyses were conducted using a Xevo G2-XS QTOF instrument (Waters Corporation, Milford, MA, USA) with electrospray ionization. The LC–MS samples were separated on an Acquity UPLC (Waters Corporation, Milford, MA, USA) C18 1.7 μm column as solutions in water or acetonitrile, prepared at 0.15–0.20 mg/mL concentration. The LC–MS chromatography was carried out with linear gradients of 0.05% formic acid in acetonitrile and 0.05% formic acid in water. ^1^H, ^13^C, and HSQC NMR spectra were obtained on a Varian VNMRS 500 or 400 instrument or a Bruker Avance Neo 500 (Bruker USA, San Jose, CA, USA). Chemical shifts (δ) are reported in parts per million (ppm) and are referenced to the residual solvent signal. Coupling constants (*J*) are reported in hertz (Hz). The usual abbreviations are used to describe multiplicities: s (singlet), d (doublet), t (triplet), q (quartet), br (broad), and app (apparent). Minor impurities such as solvents, water, and grease are not reported in the NMR transcriptions.

### 3.2. 6-Chloro-2-methylquinolin-4(1H)-one (***7***)

4-Chloroaniline **6** (3.30 g, 26.3 mmol, 1 equiv) was dissolved in 150 mL of toluene in an oven-dried round bottom flask containing a stir bar and 9 g of activated 4 Å molecular sieves. Ethyl acetoacetate (4.54 mL, 52.6 mmol, 2 equiv) and glacial acetic acid (17.5 mmol, 1.5 equiv) were added to the reaction flask at room temperature, producing a pale-yellow solution. The reaction was stirred for 48 h, at which time NMR analysis indicated the complete disappearance of the starting aniline. The molecular sieves were removed via filtration. The resulting yellow solution was concentrated, and the residue was dissolved in 45 mL of Dowtherm A and transferred with a syringe to a 2-neck oven-dried round bottom flask containing 45 mL of Dowtherm A heated at 250 °C. The reaction mixture was heated at reflux for 25 min. The flask was cooled to room temperature by using a water bath. Upon cooling, a yellow precipitate formed in the dark red solution. The precipitate (6.25 g) was collected via vacuum filtration and washed with cold propionitrile. Crystallization of the resulting yellow solid (contaminated with Dowtherm A) from 1 L of propionitrile afforded a dark yellow solid (3.10 g, 62%): mp 314–317 °C; Lit. [22] 316–318 °C; ^1^H NMR (500 MHz, DMSO-*d*_6_) δ 11.73 (br s, 1H), 7.96 (d, *J* = 2.5 Hz, 1H), 7.65 (dd, *J* = 8.5 Hz, 2.5 Hz, 1H), 7.53 (d, *J* = 9 Hz, 1H), 5.95 (s, 1 H), 2.34 (s, 3H); ^13^C NMR (125 MHz, DMSO-*d*_6_) δ 175.5, 150.3, 138.7, 131.6, 127.4, 125.5, 123.8, 120.2, 108.6, 19.5; ESI-MS [M + H]^+^ calc’d for C_10_H_9_ClNO, 194.0294; found 194.0352.

### 3.3. 6-Chloro-3-iodo-2-methylquinolin-4(1H)-one (***8***)

A suspension of quinolinone **7** (1.261 g, 6.51 mmol, 1 equiv) in acetonitrile (32.6 mL) was stirred at room temperature for 1 h. N-iodosuccinimide (2.197 g, 9.76 mmol, 1.5 equiv) was added to the reaction flask, producing a pale brown suspension within the orange supernatant. The reaction mixture was heated at reflux for 5 h. After cooling, the white precipitate (2.03 g, 98%) was collected by vacuum filtration and washed with cold acetonitrile: mp 236–238 °C; Lit. [22] 237–238 °C; ^1^H NMR (500 MHz, DMSO-*d*_6_) δ 12.31 (br s, 1H), 8.00 (d, *J* = 2.5 Hz, 1H), 7.71 (dd, *J* = 8.5 Hz, 2.5 Hz, 1H), 7.59 (d, *J* = 9 Hz, 1H), 2.63 (s, 3H); ^13^C NMR (125 MHz, DMSO-*d*_6_) δ 171.9, 151.9, 137.5, 132.1, 128.2, 124.3, 121.4, 120.2, 86.2, 26.2; ESI-MS [M + H]^+^ calcd for C_10_H_8_ClNO, 319.9333; found 319.9346.

### 3.4. 4-(Benzyloxy)-6-chloro-3-iodo-2-methylquinoline (***9***)

Iodinated quinolinone **8** (959 mg, 3 mmol, 1 equiv) and cesium carbonate (1.466 g, 4.5 mmol, 1.5 equiv) were combined in a 3-neck oven-dried round bottom flask equipped with a stir bar under a nitrogen atmosphere. Dry acetonitrile (28 mL) was added to the reaction flask, which resulted in a white, cloudy suspension. The reaction mixture was stirred for 5 h. Benzyl bromide (0.428 mL, 3.6 mmol, 1.2 equiv) was added dropwise, and the reaction was stirred for 15 h. The resulting white suspension was collected by vacuum filtration, washed sequentially with cold water and acetonitrile, and then dried under vacuum to afford **9** as an off-white solid (1.077 g, 88%): mp 110–112 °C; ^1^H NMR (500 MHz, chloroform-*d*) δ 7.96 (d, *J* = 9 Hz, 1H), 7.93 (d, *J* = 2.5 Hz, 1H), 7.64–7.60 (m, 3H), 7.48–7.41 (m, 3H), 5.15 (s, 2H), 2.97 (s, 3H); ^13^C NMR (125 MHz, chloroform-*d*) δ 162.3, 162.3, 147.6, 136.0, 132.6, 131.5, 130.7, 129.2, 129.1, 128.7, 123.6, 121.3, 91.6, 76.6, 30.9; ESI-MS [M + H]^+^ calcd for C_17_H_14_ClINO, 409.9803; found 409.9814.

### 3.5. (3S,3aR,6S,6aR)-6-(4-(4,4,5,5-Tetramethyl-1,3,2-dioxaborolan-2-yl)phenoxy)hexahydrofuro[3,2-b]furan-3-yl Acetate (***12***)

Diisopropyl azodicarboxylate (1.38 mL, 7.0 mmol, 1.6 equiv) was added to a solution of triphenylphosphine (1.84 g, 7.0 mmol, 1.6 equiv) in cyclopentyl methyl ether (60 mL) in a 100 mL 3-neck round bottom flask at 0–5 °C (ice/water) over a 2 min period under an argon atmosphere. The resulting light yellow suspension was stirred for 15 min at this temperature, and then 4-(4,4,5,5-tetramethyl-1,3,2-dioxaborolan-2-yl)phenol **10** (1.0 g, 4.5 mmol, 1 equiv) was added to the reaction mixture. After 2 min, the cooling bath was removed to allow the reaction mixture to warm to room temperature. After 15 min, 2-*O*-acetyl isosorbide **11** (1.04 g, 5.5 mmol, 1.2 equiv) was added to the reaction mixture with stirring, followed by diisopropylethylamine (1.22 mL, 7.0 mmol, 1.6 equiv). During a 30 min period, all the solids dissolved to form a light brown solution. The reaction mixture was stirred for an additional 30 min, then heated at 90–95 °C for 5 days until TLC analysis indicated the complete disappearance of the starting material. The brown solution was cooled to room temperature and concentrated in vacuo. The residue was chromatographed with 4:1 hexane/ethyl acetate as an eluant to produce **12** (R_f_ = 0.25) as a viscous yellow oil (1.47 g, 83%). ^1^H NMR (500 MHz, chloroform-*d*) δ 7.70 (br d, *J* = 8.65 Hz, 2H), 6.86 (br d, *J* = 8.70 Hz, 2 H), 5.16 (d, *J* = 3.65 Hz, 1H), 4.80 (br t, *J* = 2.68 Hz, 1 H), 4.68 (d, *J* = 3.85 Hz, 1H), 4.63 (d, *J* = 3.85, 1 H), 4.01–4.00 (m, 2H), 3.97 (dd, *J* = 10.65 Hz, 3.80 Hz, 1H), 3.87 (br d, *J* = 10.60 Hz, 1H), 2.00 (s, 3H), 1.27 (s, 12H); ^13^C NMR (125 MHz, chloroform-*d*) δ 170.3, 159.7, 137.0, 122.0, 114.8, 85.8, 85.8, 84.0, 81.0, 77.9, 72.86, 72.78, 25.2, 21.2; HR-LC–ESI-MS [M + H]^+^ *m*/*z* calcd for C_20_H_27_BO_7_, 391.1922; found, 391.1941.

### 3.6. (3S,3aR,6S,6aR)-6-(4-(4-(Benzyloxy)-6-chloro-2-methylquinolin-3-yl)phenoxy)hexahydrofuro[3,2-b]furan-3-yl Acetate (***13***)

Protected iodoquinoline **9** (437.4 mg, 1.1 mmol, 1 equiv) and Pd(dppf)Cl_2_ (39.1 mg, 0.053 mmol, 0.05 equiv) were combined in a 3-neck round-bottom flask equipped with a stir bar under a nitrogen atmosphere. A solution of phenyl boronic ester isosorbide acetate **12** (500 mg, 1.28 mmol, 1.2 equiv) in 8 mL of DMF under a nitrogen atmosphere was added to the reaction flask, producing an orange–red suspension. Aqueous potassium carbonate (2 N, 2.14 mL, 2.14 mmol, 2 equiv) was added dropwise, leading to the formation of a fluffy white precipitate in the red solution. The reaction was slowly heated to 85 °C and stirred for 15 min until TLC analysis indicated the disappearance of the starting material. The reaction mixture was cooled to room temperature and then decanted into a separatory funnel containing 20 mL of ethyl acetate. The organic layer was washed with saturated aqueous ammonium chloride (3 X 7 mL), dried over anhydrous sodium sulfate, and then concentrated in vacuo. The residue was chromatographed with 5:2 hexane/ethyl acetate as an eluant to produce **13** (R_f_ = 0.33) as a white sticky solid (402 mg, 69%): mp 51–55 °C; ^1^H NMR (500 MHz, acetonitrile-*d_3_*) δ 8.05 (d, *J* = 2.4 Hz, 1H), 7.93 (d, *J* = 8.95 Hz, 1H), 7.65 (dd, *J* = 8.9 Hz, 2.4 Hz, 1H), 7.37 (d, *J* = 8.75 Hz, 2H), 7.31–7.28 (m, 3H), 7.12–7.09 (m, 4H), 5.15 (d, *J* = 3.65 Hz, 1H), 4.94 (d, *J* = 3.2 Hz, 1H), 4.73 (d, *J* = 4.05 Hz, 1H), 4.68 (m, 3H), 4.09 (dd, *J* = 10.45 Hz, 3.9 Hz, 1H), 4.04–3.99 (m, 2H), 3.91 (d, *J* = 10.7 Hz, 1H), 2.45 (s, 3H), 2.03 (s, 3H); ^13^C NMR (125 MHz, acetonitrile-*d_3_*) δ 170.9, 161.6, 159.4, 157.8, 147.8, 137.5, 132.6, 131.9, 131.5, 131.0, 129.4, 129.38, 129.33, 129.31, 128.1, 124.7, 122.4, 116.6, 86.5, 86.4, 82.3, 78.6, 76.6, 72.9, 25.2, 21.1 (one carbon in the aromatic region is unaccounted for); HR-LC-ESI–MS [M + H]^+^ *m*/*z* calcd for C_31_H_29_ClNO_6_, 546.1677; found, 546.1700.

### 3.7. (3S,3aR,6S,6aR)-6-(4-(6-Chloro-2-methyl-4-oxo-1,4-dihydroquinolin-3-yl)phenoxy)hexahydrofuro[3,2-b]furan-3-yl Acetate (***14***)

Acetylated Suzuki coupling product **13** (95 mg, 0.174 mmol, 1 equiv) was dissolved in 3 mL of dry acetonitrile in an oven-dried 3-neck round bottom flask equipped with a stir bar under a nitrogen atmosphere. Sodium iodide (45 mg, 0.300 mmol, 1.7 equiv) and trimethylsilyl chloride (26 μL, 0.211 mmol, 1.2 equiv) were added to the reaction flask, producing a pale-yellow suspension. The reaction was stirred for 8 h at room temperature, whereupon the starting material was consumed according to the TLC analysis. The resulting white suspension was filtered, and the precipitate was washed sequentially with cold water and acetonitrile and then dried under vacuum, affording **14** as an off-white solid (56 mg, 70%): mp 298–299 °C; ^1^H NMR (500 MHz, DMSO-*d*_6_) δ 11.78 (br s, 1H), 7.98 (d, *J* = 2 Hz, 1H), 7.64 (dd, *J* = 8.85 Hz, 2.2 Hz, 1H), 7.55 (d, *J* = 8.85 Hz, 1H), 7.16 (d, *J* = 8.25 Hz, 2H), 6.97 (d, *J* = 8.3 Hz, 2H), 5.06 (d, *J* = 3.25 Hz, 1H), 4.90 (d, *J* = 2 Hz, 1H), 4.64 (d, *J* = 4.1 Hz, 1H), 4.61 (d, *J* = 4.1 Hz, 1H), 4.03 (dd, *J* = 10.4 Hz, 3.95 Hz, 1H), 3.95 (dd, *J* = 10.65 Hz, 3.7 Hz, 1H), 3.91 (d, *J* = 10.25 Hz, 1H), 3.85 (d, *J* = 10.65 Hz, 1H), 2.21 (s, 3H), 2.01 (s, 3H); ^13^C NMR (125 MHz, DMSO-*d*_6_) δ 174.0, 169.9, 155.5, 147.4, 138.0, 132.3, 131.7, 128.6, 127.5, 125.4, 124.4, 120.7, 120.2, 114.8, 85.1, 85.1, 80.8, 77.3, 71.8, 71.6, 20.8, 19.1; HR-LC–ESI-MS [M + H]^+^ *m*/*z* calcd for C_24_H_23_ClNO_6_, 456.1208; found, 456.1219.

### 3.8. 6-Chloro-3-(4-(((3S,3aR,6S,6aR)-6-hydroxyhexahydrofuro[3,2-b]furan-3-yl)oxy)phenyl)-2-methylquinolin-4(1H)-one (***15***)

Acetylated Suzuki coupling product **13** (100 mg, 0.219 mmol, 1 equiv) was dissolved in 6 mL of dioxane to form a pale-yellow solution. Aqueous potassium hydroxide (1.0 M, 3 mL) was slowly added to the reaction mixture, and the reaction was stirred for 30 min, whereupon TLC analysis indicated the disappearance of the starting material. The solution was concentrated, and the residue was suspended in 12 mL of deionized water and transferred to a separatory funnel. The aqueous layer was extracted with dichloromethane (3 × 5 mL), and the combined extract was dried over anhydrous sodium sulfate and then concentrated to provide the alcohol as a white solid (90.1 mg, 99%): mp 179.5–180 °C; ^1^H NMR (500 MHz, chloroform-*d*) δ 8.04 (d, *J* = 2.3 Hz, 1H), 7.95 (d, *J* = 8.95 Hz, 1H), 7.6 (dd, *J* = 8.95 Hz, 2.35 Hz, 1H), 7.33–7.29 (m, 5H), 7.10–7.06 Hz, (m, 4H), 4.87–4.86 (m, 2H), 4.68 (d, *J* = 3.75 Hz, 1H), 4.62 (s, 2H), 4.44 (br s, 1H), 4.13–4.08 (m, 2H), 3.98 (dd, *J* = 10.1 Hz, 3.3 Hz, 1 H), 3.93 (d, *J* = 10 Hz, 1H), 3.02 (s, 3H); ^13^C NMR (125 MHz, chloroform-*d*) δ 160.5, 158.9, 156.9, 146.9, 136.3, 131.7, 130.7, 130.2, 128.6, 128.55, 128.51, 128.3, 126.5, 123.8, 121.7, 115.7, 88.2, 85.4, 81.4, 76.1, 75.8, 74.8, 72.5, 25.1 (one isosorbide carbon is unaccounted for); HR-LC–ESI-MS [M + H]^+^ *m*/*z* calcd for C_29_H_27_ClNO_5_, 504.1572; found, 504.1581.

The alcohol from above (20 mg, 0.040 mmol, 1 equiv) was dissolved in 2 mL of glacial acetic acid to form a pale-yellow solution. Aqueous hydrochloric acid (1 M, 2 mL, 2 mmol, 50 equiv) was added slowly. The resulting pale-yellow solution was slowly heated to 55 °C and then stirred for 6 h, whereupon TLC analysis indicated the disappearance of the starting material. The solvent was removed under vacuum, and the yellow residue was suspended in 1 mL of acetonitrile, leading to the separation of the solid product. The solution was decanted, and the remaining precipitate was dried under vacuum to afford **15** as an off-white solid (14 mg, 85%); mp 257 °C (dec); ^1^H NMR (500 MHz, DMSO-*d*_6_) δ 8.0 (br s, 1H), 7.67–7.62 (m, 2H), 7.18 (d, *J* = 8 Hz, 2H), 6.99 (d, *J* = 8 Hz, 2H), 5.29 (br s, 1H), 4.86 (d, *J* = 2 Hz, 1H), 4.60 (d, *J* = 3.5 Hz, 1H), 4.45 (d, *J* = 3 Hz, 1H), 4.13 (br s, 1H), 3.98 (dd, *J* = 10.5 Hz, 2.5 Hz, 1 H), 3.88 (d, *J* = 10 Hz, 1H), 3.80 (dd, *J* = 9 Hz, 3 Hz, 1H), 3.70 (d, *J* = 9.5 Hz, 1H), 2.24 (s, 3H); ^13^C NMR (125 MHz, DMSO-*d*_6_) δ 174.0, 155.5, 147.6, 138.0, 132.3, 131.5, 128.5, 127.4, 125.3, 124.3, 120.7, 120.3, 114.8, 87.8, 84.8, 80.9, 74.7, 74.3, 71.5, 18.9; HR-LC–ESI-MS [M + H]^+^ *m*/*z* calcd for C_29_H_27_ClNO_5_, 504.1572; found, 504.1581.

### 3.9. 6-Chloro-3-(4-(((3S,3aR,6S,6aR)-6-methoxyhexahydrofuro[3,2-b]furan-3-yl)oxy)phenyl)-2-methylquinolin-4(1H)-one (***16***)

The deacetylated Suzuki coupling product, prepared as above (100 mg, 0.200 mmol, 1 equiv), was dissolved in 4 mL of dichloromethane under an argon atmosphere. Tetra-*n*-butylammonium bromide (19.8 mg, 0.061 mmol, 0.31 equiv) was added to the reaction mixture, followed by 1 mL of an aqueous 50% potassium hydroxide solution. Dimethyl sulfate (25 µL, 0.264 mmol, 1.32 equiv) was added dropwise to the resulting pale-yellow solution. The reaction was stirred for 2.5 h until TLC analysis indicated the complete disappearance of the starting material. The reaction mixture was decanted into the separatory funnel containing 6 mL of dichloromethane and washed with saturated aqueous ammonium chloride (2 × 5 mL). The combined organic layer was dried over anhydrous sodium sulfate and concentrated in vacuo. The residue was chromatographed with 3:2 hexane/ethyl acetate as the eluant to produce the protected methyl ether (R_f_ = 0.38) as sticky colorless crystals (79.8 mg, 78%): mp 38–41 °C; ^1^H NMR (500 MHz, acetonitrile-*d_3_*) δ 8.05 (d, *J* = 2.35 Hz, 1H), 7.92 (d, *J* = 8.95 Hz, 1H), 7.65 (dd, *J* = 8.95 Hz, 2.4 Hz, 1H), 7.37 (d, *J* = 8.8 Hz, 2H), 7.30–7.28 (m, 3H), 7.12–7.08 (m, 4H), 4.89 (d, *J* = 2.25 Hz, 1H), 4.67 (m, 3H), 4.65 (d, *J* = 4.2 Hz, 1H), 4.05 (dd, *J* = 10.4 Hz, 3.95 Hz, 1H), 4.00 (dd, *J* = 10.35 Hz, 1.7 Hz, 1H), 3.90–3.84 (m, 3 H), 3.36 (s, 3H), 2.45 (s, 3H); ^13^C NMR (125 MHz, acetonitrile-*d_3_*) δ 161.6, 159.4, 157.8, 147.7, 137.4, 132.5, 131.9, 131.4, 131.0, 129.3, 129.29, 129.2, 128.0, 124.7, 122.4, 118.3, 116.5, 86.3, 86.2, 85.9, 82.4, 76.6, 72.6, 72.59, 57.4, 25.2; HR-LC–ESI-MS [M + H]^+^ *m*/*z* calcd for C_30_H_29_ClNO_5_, 518.1728; found, 518.1746.

The protected methyl ether from above (19.5 mg, 0.038 mmol, 1 equiv) was dissolved in 2 mL of glacial acetic acid to form a pale-yellow solution. Aqueous hydrochloric acid (1 M, 2 mL, 2 mmol, 52 equiv) was slowly added at room temperature. The resulting solution was slowly heated to 55 °C and stirred for 6 h, whereupon TLC analysis indicated the disappearance of the starting material. The solvent was removed under vacuum, and the yellow residue was suspended in 1 mL of acetonitrile, leading to the separation of the solid product. The solution was decanted, and the precipitate was dried under vacuum to afford **16** as an off-white solid (14.5 mg, 90%): mp 262 °C (dec); ^1^H NMR (500 MHz, DMSO-*d*_6_) δ 8.01 (br s, 1H), 7.67 (app br s, 2H), 7.18 (d, *J* = 8.6 Hz, 2H), 6.99 (d, *J* = 8.65 Hz, 2H), 4.87 (br s, 1H), 4.63 (d, *J* = 4.15 Hz, 1H), 4.56 (d, *J* = 4.15 Hz, 1H), 4.01 (dd, *J* = 10.35 Hz, 4.15 Hz, 1H), 3.90 (app br d, *J* = 12.05 Hz, 2H), 3.85–3.80 (m, 2H), 3.30 (s, 3H), 2.25 (s, 3H); ^1^C NMR (125 MHz, DMSO-*d*_6_) δ 173.7, 155.5, 147.4, 137.9, 132.2, 131.5, 128.5, 127.3, 125.3, 124.2, 120.6, 120.2, 114.7, 85.0, 84.8, 84.3, 80.8, 71.4, 71.2, 56.5, 18.9; HR-LC–ESI-MS [M + H]^+^ *m*/*z* calcd for C_23_H_23_ClNO_5_, 428.1259; found, 428.1291.

### 3.10. 6-Chloro-3-(4-(((3S,3aR,6S,6aR)-6-(2-methoxyethoxy)hexahydrofuro[3,2-b]furan-3-yl)oxy)phenyl)-2-methylquinolin-4(1H)-one (***17***)

The deacetylated Suzuki coupling product prepared as above (110 mg, 0.218 mmol, 1 equiv) was dissolved in 5 mL of dry DMF under an argon atmosphere. Sodium hydride (52 mg, 2.17 mmol, 9.9 equiv) was added to the reaction flask to produce a light brown suspension. The reaction was stirred at room temperature for 40 min, and then a solution of bromoethyl methyl ether (0.12 mL, 1.28 mmol, 5.9 equiv) in 0.5 mL of dry DMF was added dropwise. The reaction was stirred for an additional 8 h until TLC analysis indicated the complete disappearance of the starting material. The light brown suspension was cooled to room temperature and concentrated in vacuo. The residue was chromatographed with 2:1 hexane/ethyl acetate as the eluant to produce the protected methoxyethyl ether (R_f_ = 0.33) as a colorless oil (91.4 mg, 75%). ^1^H NMR (500 MHz, chloroform-*d*) δ 8.04 (d, *J* = 2.3 Hz, 1H), 7.96 (d, *J* = 8.95, 1H), 7.61 (dd, *J* = 8.95 Hz, 2.4 Hz, 1H), 7.32–7.28 (m, 5H), 7.11–7.07 (m, 2H), 7.06 (d, *J* = 8.7Hz, 2H), 4.86 (br d, *J* = 2.65 Hz, 1H), 4.80 (d, *J* = 4.05 Hz, 1H), 4.77 (d, *J* = 4.05 Hz, 1H), 4.62 (s, 2H), 4.14–4.08 (m, 3H), 4.00–3.95 (m, 2H), 3.75–3.66 (m, 2H), 3.55 (t, *J* = 9.3 Hz, 4.6 Hz, 2H), 3.38 (s, 3H), 2.52 (s, 3H); ^13^C NMR (125 MHz, chloroform-*d*) δ160.5, 158.9, 156.9, 146.9, 136.3, 131.7, 130.6, 130.3, 128.6, 128.5, 128.47, 128.4, 128.3, 126.5, 123.7, 121.7, 115.6, 86.0, 85.5, 84.1, 81.4, 75.8, 72.5, 72.3, 72.0, 69.3, 59.3, 25.1; HR-LC–ESI-MS [M + H]^+^ *m*/*z* calcd for C_32_H_33_ClNO_6_, 562.1990; found, 562.2001.

The O-alkylated Suzuki coupling product from above (35.6 mg, 0.063 mmol, 1 equiv) was dissolved in 2 mL of glacial acetic acid. Aqueous hydrochloric acid (1 M, 4 mL, 4 mmol, 63.5 equiv) was slowly added at room temperature. The resulting pale-yellow solution was heated to 55 °C and stirred for 24 h at that temperature, whereupon TLC analysis indicated the disappearance of the starting material. The solvent was removed under vacuum, and the yellow residue was suspended in 1 mL of acetonitrile, leading to the separation of the solid product. The solution was decanted, and the precipitate was dried under vacuum to afford **17** as an off-white solid (26.6 mg, 89%): mp 185 °C (dec); ^1^H NMR (500 MHz, DMSO-*d*_6_, 18 °C) δ 8.13 (d, *J* = 2.25 Hz, 1H), 7.86 (d, *J* = 8.9 Hz, 1H), 7.73 (dd, *J* = 8.85 Hz, 2.3 Hz, 1H), 7.20 (d, *J* = 8.55 Hz, 2H), 7.00 (d, *J* = 8.55 Hz, 2H), 4.88 (s, 1H), 4.62 (d, *J* = 4.1 Hz, 1H), 4.56 (d, *J* = 4.05 Hz, 1H), 4.01–3.98 (m, 3H), 3.90 (d, *J* = 10.2 Hz, 1H), 3.84–3.80 (m, 2H), 3.62–3.56 (m, 2H), 3.42 (t, *J* = 9.35 Hz, 4.65 Hz, 2H), 3.23 (s, 3H), 2.30 (s, 3H); ^13^C NMR (125 MHz, DMSO-*d*_6_) δ 172.3, 155.7, 149.0, 137.9, 132.1, 131.8, 128.0, 127.9, 124.5, 124.1, 120.6, 120.5, 114.9, 85.2, 85.0, 83.1, 80.8, 71.7, 71.4, 71.2, 68.1, 58.1, 19.0; HR-LC–ESI-MS [M + H]^+^ *m*/*z* calcd for C_25_H_27_ClNO_6_, 472.1521; found, 472.1529.

### 3.11. In Vitro Screening

The ELQs **14**–**17** were tested against the chloroquine-sensitive *Plasmodium falciparum* strain 3D7 (MRA-102), as provided by the MR4 Unit of the American Type Culture Collection (ATCC, Manassas, VA, USA). Details of the assay have been previously published [20]. Cytotoxicity IC_50_ values for **14**–**17** were obtained on human BJ fibroblasts as previously described [23].

### 3.12. Kinetic Solubilities

The ELQs **14**–**17** were evaluated for solubility in pH 7.4 phosphate buffer at 23 °C over a 20 h period according to the method [24] described by AstraZeneca’s Mechanistic Biology and Profiling, Discovery Sciences, R&D group (AstraZeneca, Gothenburg, Sweden).

## 4. Conclusions

The structural modification of the antimalarial ELQ scaffold, wherein the distal aryl substituent is replaced by an isoidide unit (**14**–**17**), has been carried out as a means to improve the drug-likeness qualities of molecules related to **1** and **2**. In the course of the syntheses, several modifications to previous methods for assembling ELQs were introduced: a milder method for Conrad–Limpach quinolone synthesis, the use of O-benzyl as a versatile quinolone protecting group, and the use of cyclopentyl methyl ether as a solvent for the slow and water/peroxide-sensitive Mitsunobu reaction. True to expectations, the melting points of the new analogs are reduced relative to **2**, and the aqueous solubilities are greatly improved (Table 1). While cytotoxicity levels of **14**–**17** are acceptable, the antimalarial potency of the new analogs has been lost, undoubtedly due to the hydrophilic nature of the new substituents. Even the option of modifying the terminal hydroxyl to give *O*-alkyl derivatives (**16** and **17**) did not lead to active compounds. For modifications of this nature to succeed, it will be necessary to attach the solubilizing group at a different position on the ELQ scaffold, or else more distant from the quinolone ring.

## Figures and Tables

**Table 1:** Evaluation of ELQs.

Compound	MW (g/mol)	ClogP ^a^	Melting Point (°C)	EC_50_ (nM) ^b^	Kinetic Solubility (µM) ^c^	Cytotox. IC_50_ (µM) ^d^
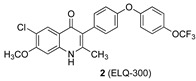	475.8	5.75	308–310 (dec)	2.2 (D6) 2.5 (Dd2) 1.4 (Tm90-C2B)	16	>10
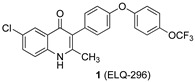	445.8	5.98	not reported	3.2 (D6) 5.6 (Dd2) 2.3 (Tm90-C2B)	not found	>10
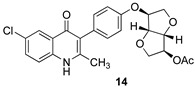	455.9	2.41	298–299	>1000	partial hydrolysis at pH 7.4	>100
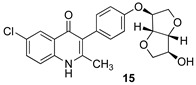	413.8	1.55	257 (dec)	>1000	95	>100
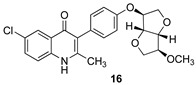	427.9	2.35	262 (dec)	>1000	22	>100
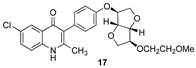	471.9	2.17	185 (dec)	>1000	127	>100

^a^ ClogP values from ChemDraw 23.1. ^b^ EC_50_ values for **1** and **2** are taken from [1]; EC_50_ measurements for **14**–**17** showed no potency below 1 millimolar when evaluated against the chloroquine-sensitive *P. falciparum* strain 3D7 according to methods described [20]. ^c^ Kinetic solubility: For **2**, see [2]; for **14**–**17**, analysis was performed by quantitative LC–MS on solutions in pH 7.4 phosphate buffer at 23 °C over a 20 h period (**14** was partially hydrolyzed to **15** under these conditions). ^d^ Cytotoxicity assays (IC_50_ values) for ELQs for **1** and **2** (see [1]) were carried out with human hepatoma-derived HepG2 cells; IC_50_ values for **14**–**17** were obtained on human BJ fibroblasts after 72 h exposure at 37 °C, using fluorescence visualization with CTG reagent.

## Data Availability

The original contributions presented in the study are included in the article/Supplementary Materials; further inquiries can be directed to the corresponding author.

## Supplementary Materials

The following supporting information can be downloaded at: https://www.mdpi.com/article/10.3390/molecules29153615/s1. Supporting Information: scanned ^1^H-NMR and ^13^C-NMR spectra for **7**–**9** and **12**–**17**.
