# Supplementary material for: Incorporation of an Isohexide Subunit into the Endochin-like Quinolone Scaffold
Source: Molecules. 2024 Jul 31;29(15):3615. doi: 10.3390/molecules29153615 (PMC11314205; doi:10.3390/molecules29153615)
Supplement: Supplementary file 1 [file molecules-29-03615-s001.zip › molecules-3084522-supplementary.pdf]

Supporting Information

Incorporation of an Isohexide Subunit into the Endochin-like Quinolone Scaffold

Julia Senkina and Spencer Knapp*

Department of Chemistry and Chemical Biology, Rutgers, The State University of New Jersey, 123 Bevier Road, Piscataway, NJ 08854, USA

Index

Scanned Spectra:	Page
^{1}H NMR and ^{13}C NMR spectra for **7**	SI – 2
^{1}H NMR and ^{13}C NMR spectra for **8**	SI – 3
^{1}H NMR and ^{13}C NMR spectra for **9**	SI – 4
^{1}H NMR and ^{13}C NMR spectra for **12**	SI – 5
^{1}H NMR and ^{13}C NMR spectra for **13**	SI – 6
^{1}H NMR and ^{13}C NMR spectra for **14**	SI – 7
^{1}H NMR and ^{13}C NMR spectra for **15**	SI – 8
^{1}H NMR and ^{13}C NMR spectra for **16**	SI – 9
^{1}H NMR and ^{13}C NMR spectra for **17**	SI – 10

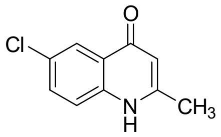
O
Cl
N
H
CH3

7

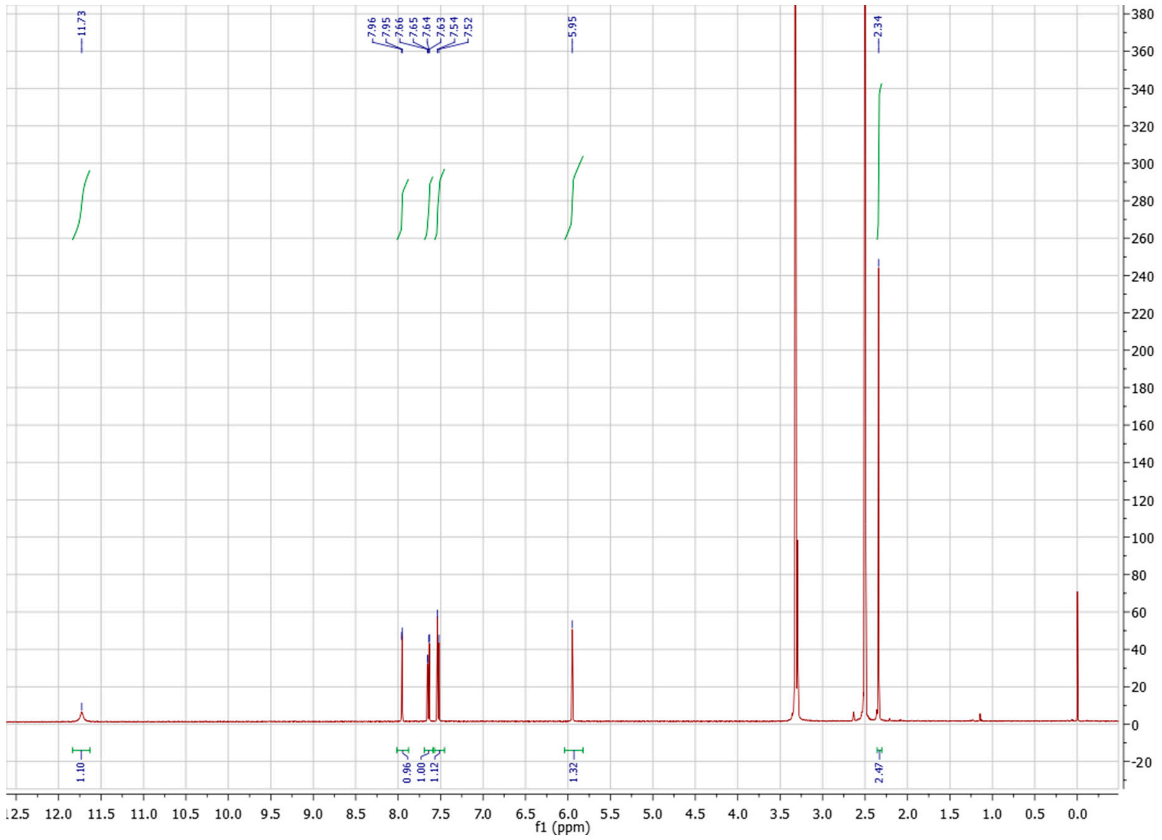
11.73
7.96
7.95
7.66
7.65
7.64
7.63
7.54
7.52
5.95
2.34
1.10
0.96
1.00
1.12
1.32
2.47
f1 (ppm)

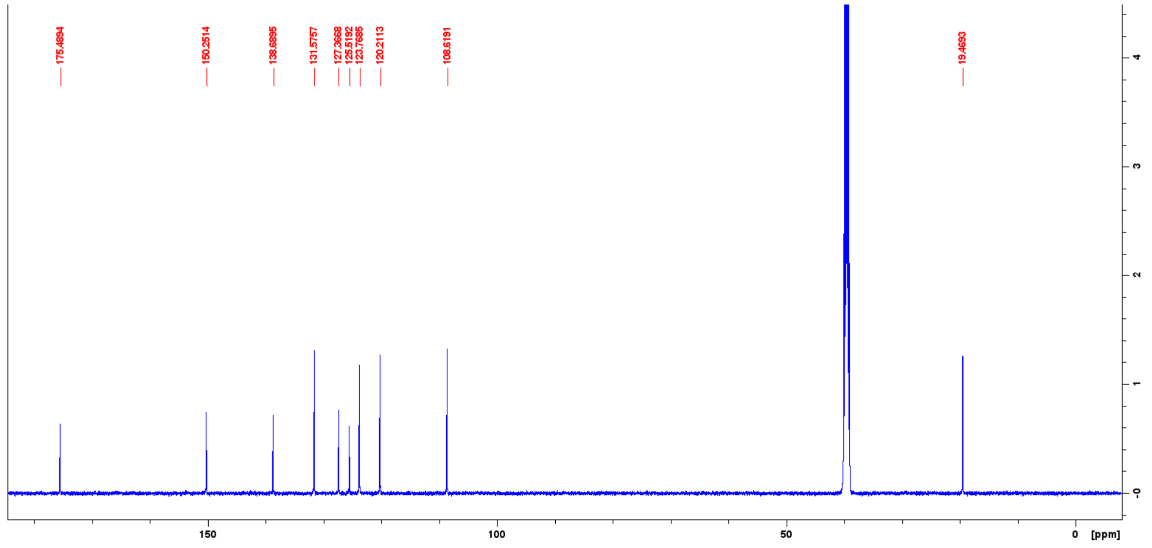
175.4894
150.2514
138.6895
131.5757
127.3668
125.5192
123.7685
120.2113
108.6191
19.4693
[ppm]

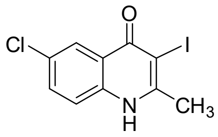
O
Cl
I
N
H
CH3

8

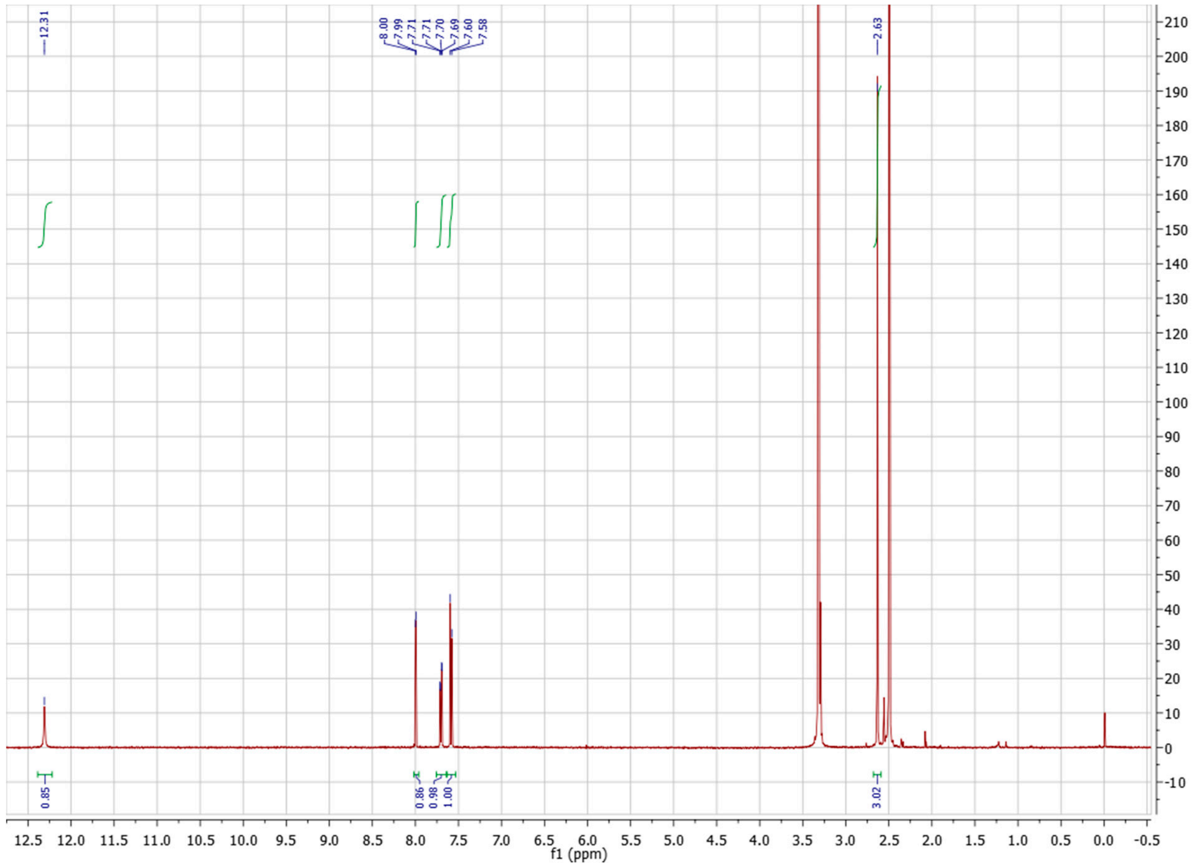
12.31
8.00
7.99
7.71
7.71
7.70
7.69
7.60
7.58
2.63
0.85
0.86
0.98
1.00
3.02
f1 (ppm)

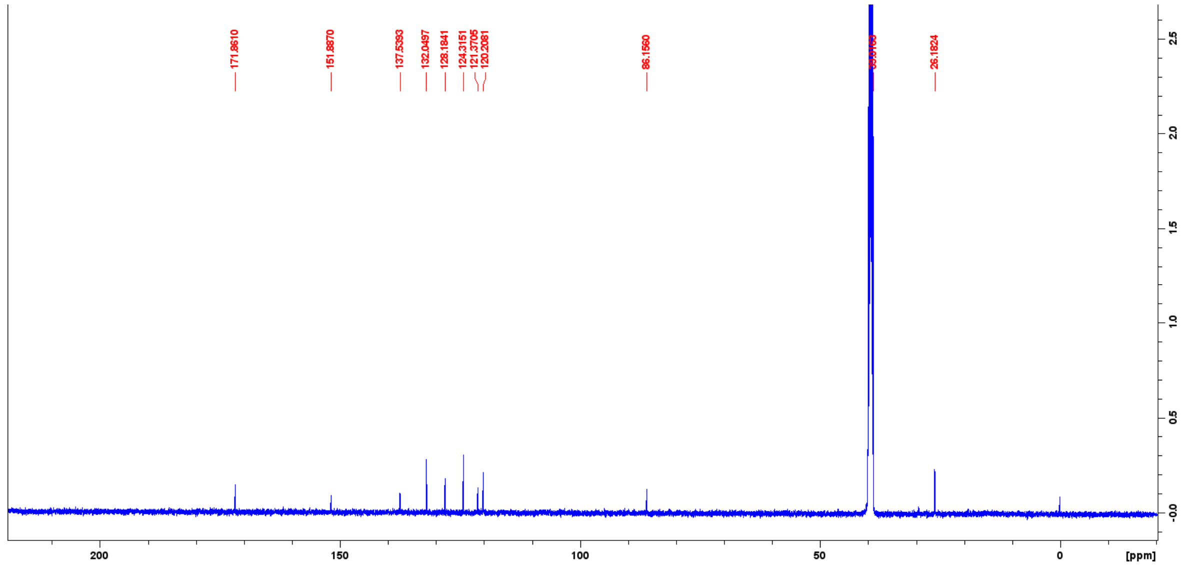
171.8610
151.8870
137.5393
132.0497
128.1841
124.3151
121.3705
120.2081
86.1560
26.1824
[ppm]

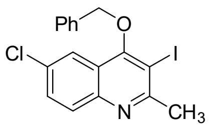

9

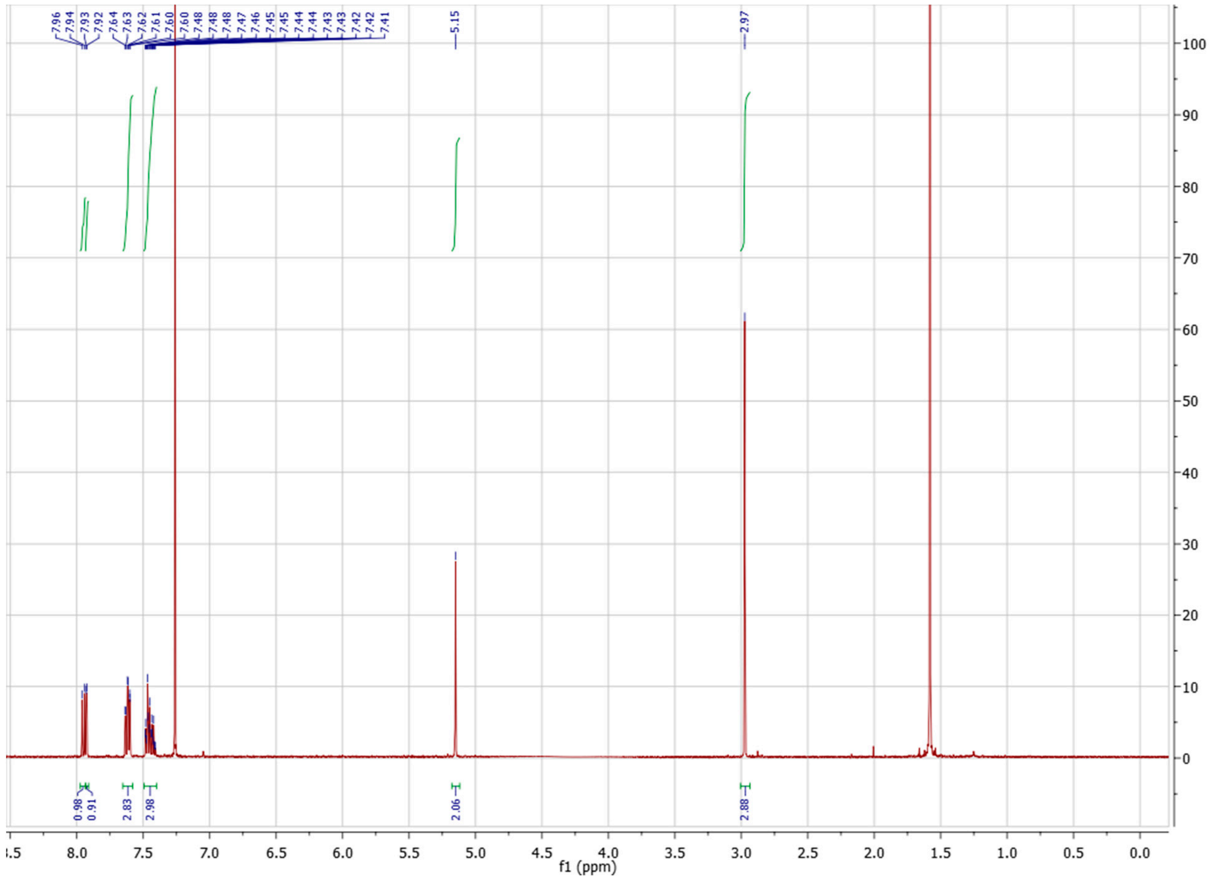

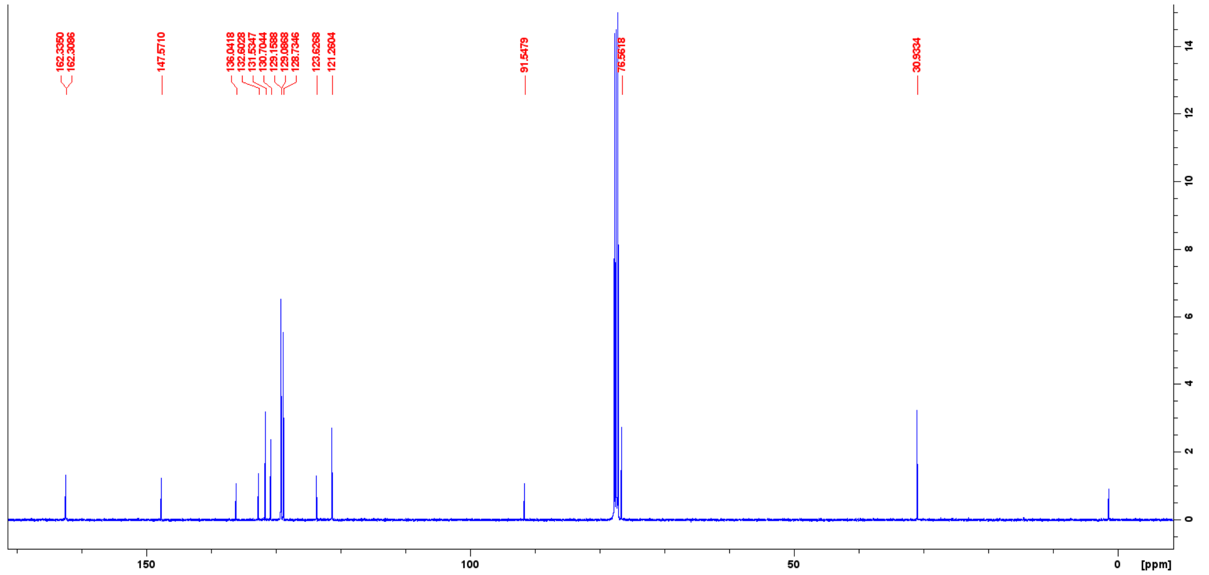

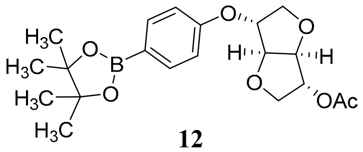

12

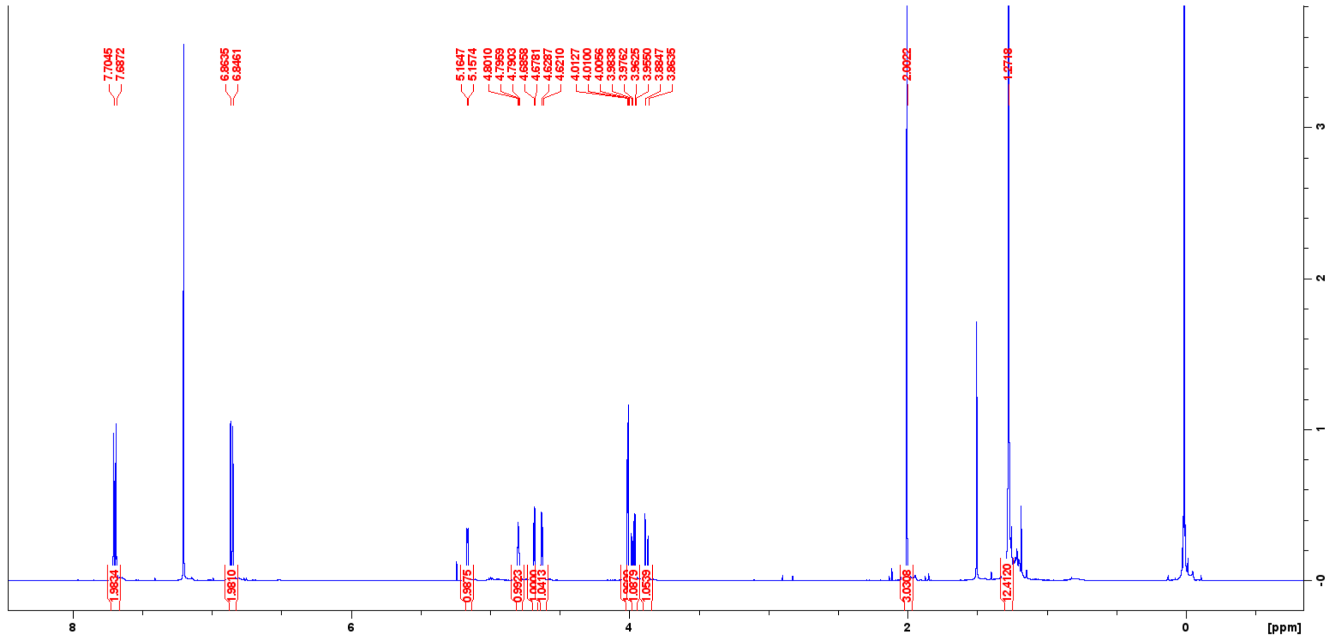

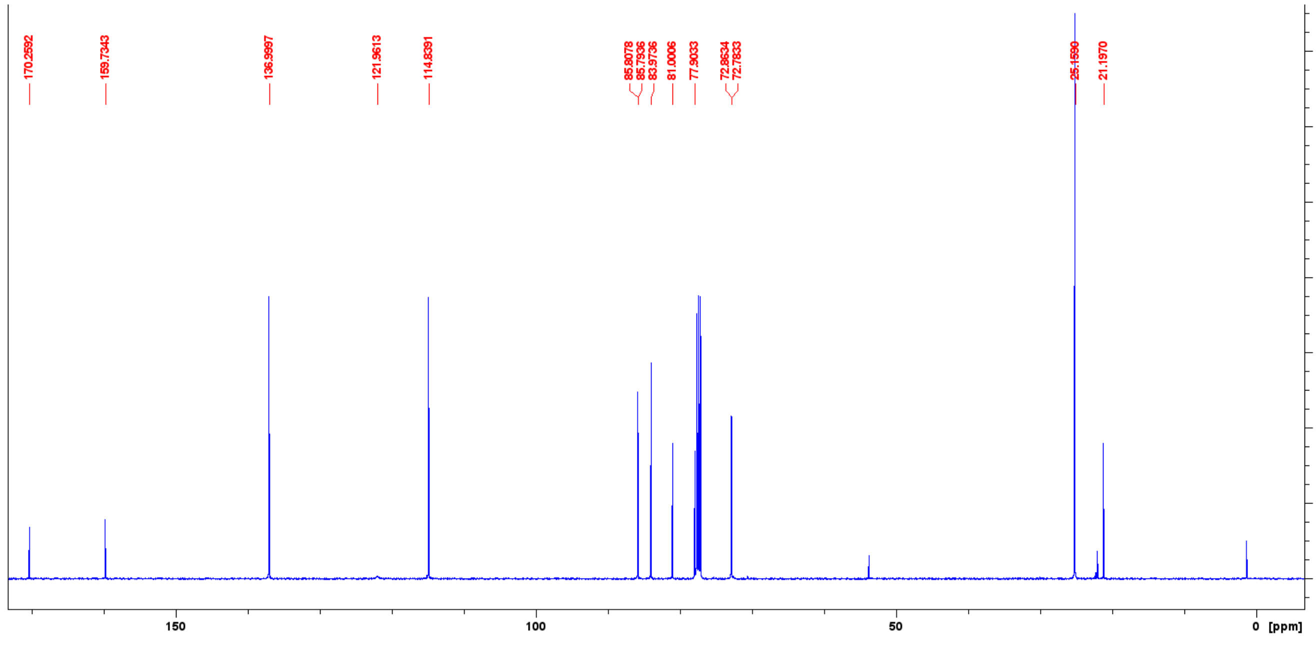

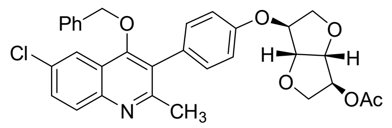

Ph
O
Cl
N
CH3
O
O
H
H
O
O
OAc

13

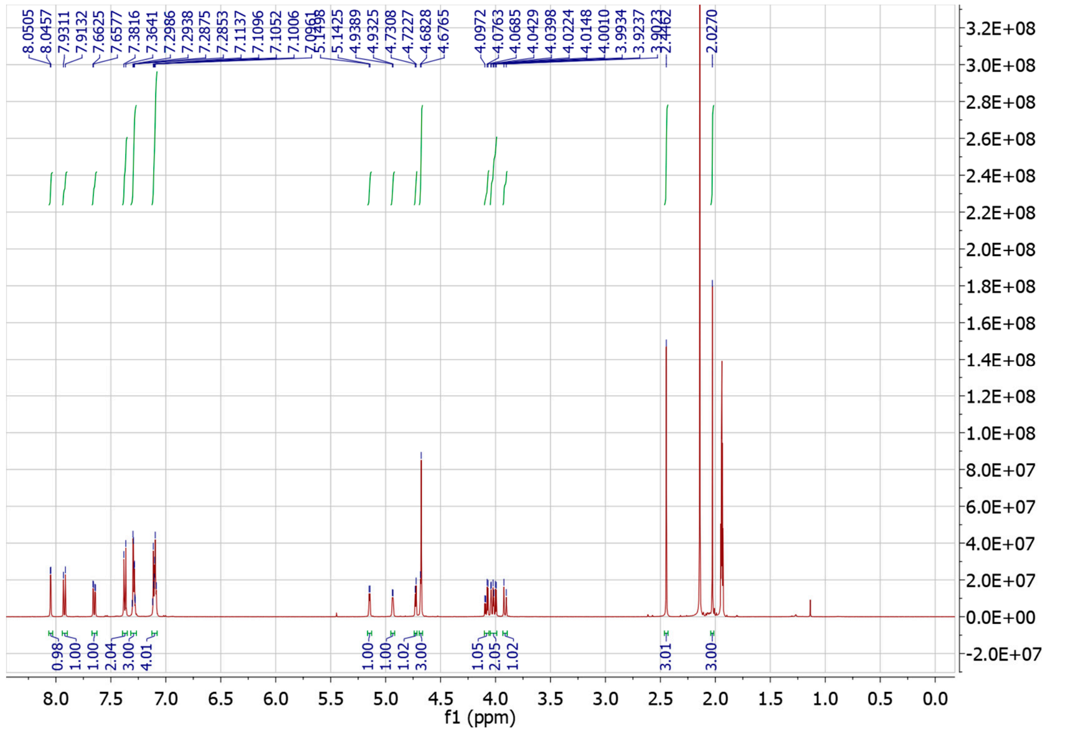

8.0505
8.0457
7.9311
7.9132
7.6625
7.6577
7.3816
7.3641
7.2986
7.2938
7.2875
7.2853
7.1137
7.1096
7.1052
7.1006
5.1425
4.9389
4.9325
4.7308
4.7227
4.6828
4.6765
4.0972
4.0763
4.0685
4.0429
4.0398
4.0224
4.0148
4.0010
3.9934
3.9237
2.0270
0.98
1.00
1.00
2.04
3.00
4.01
1.00
1.00
1.02
3.00
1.05
2.05
1.02
3.01
3.00
f1 (ppm)

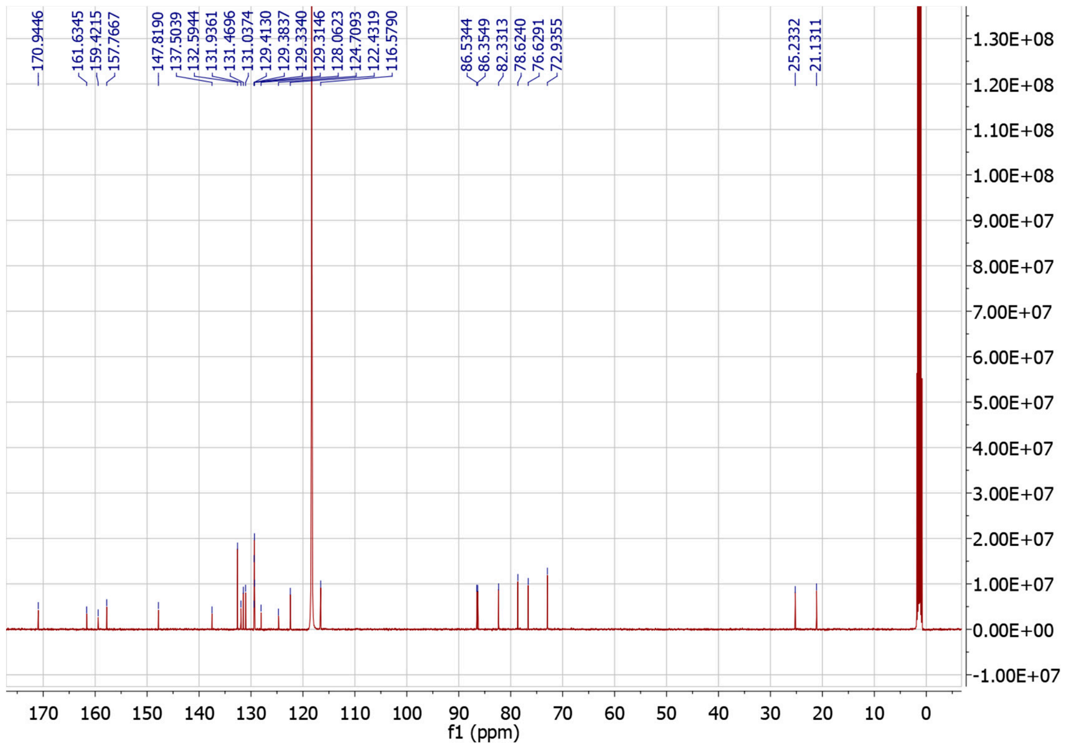

170.9446
161.6345
159.4215
157.7667
147.8190
137.5039
132.5944
131.9361
131.4696
131.0374
129.4130
129.3837
129.3340
129.3146
128.0623
124.7093
122.4319
116.5790
86.5344
86.3549
82.3313
78.6240
76.6291
72.9355
25.2332
21.1311
f1 (ppm)

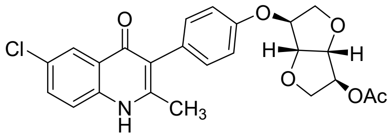

14

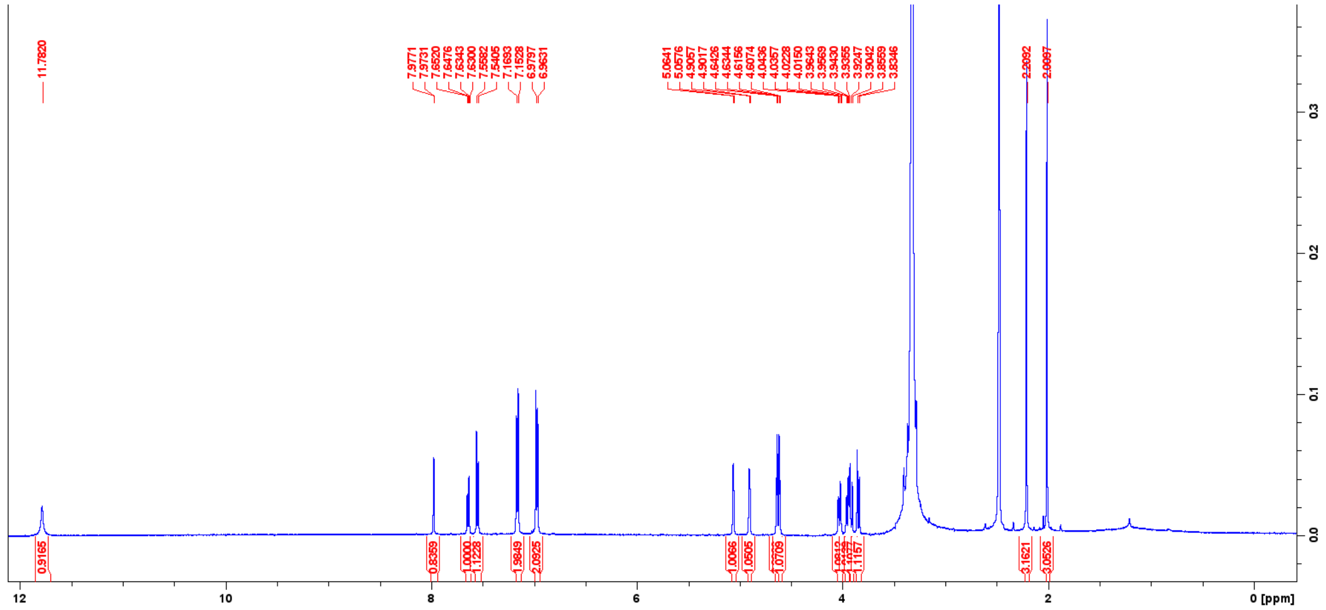

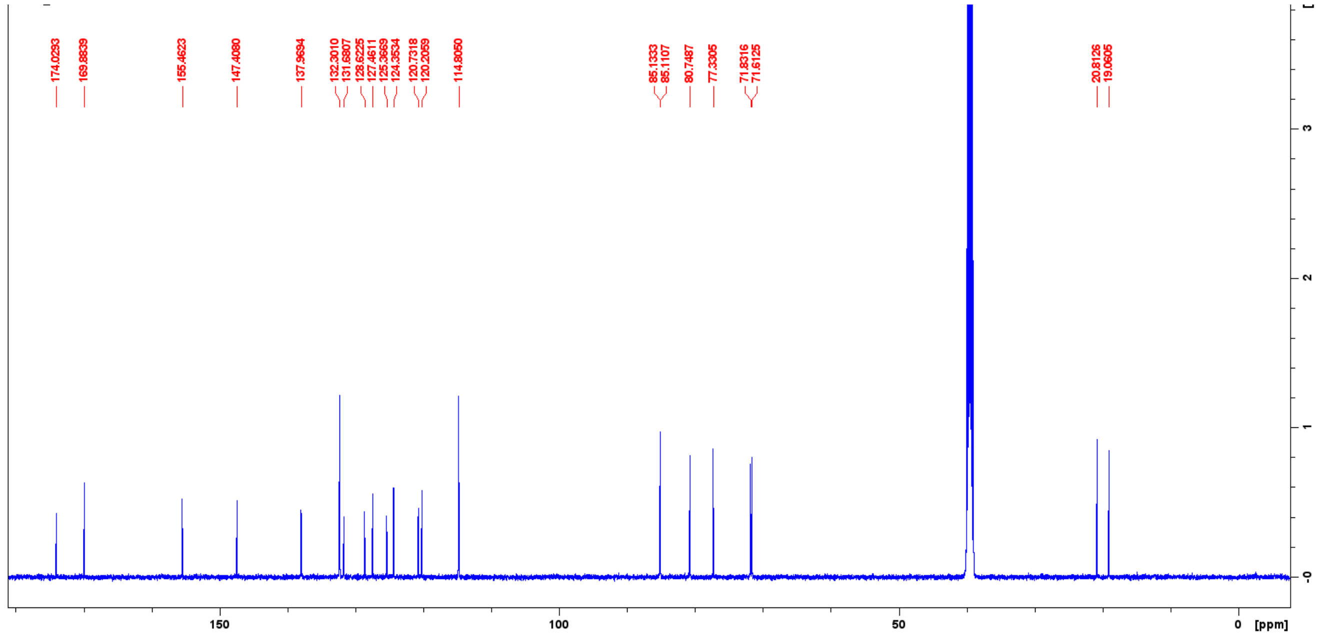

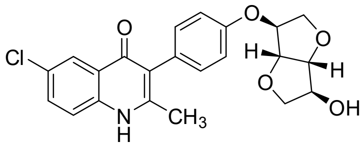
Cl
O
O
H
O
H
O
OH
N
H
CH3

15

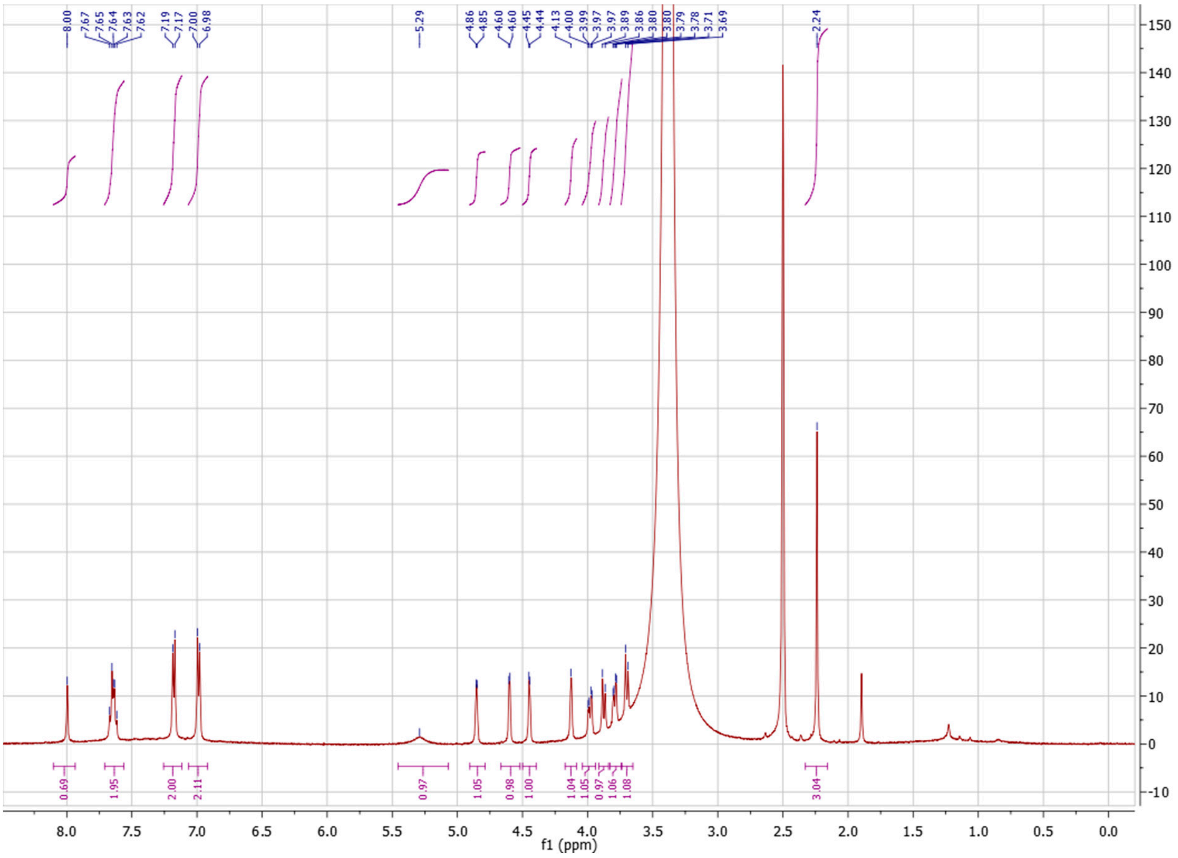
8.00
7.67
7.65
7.64
7.63
7.62
7.19
7.17
7.00
6.98
5.29
4.86
4.85
4.60
4.60
4.45
4.44
4.13
4.00
3.99
3.97
3.97
3.89
3.86
3.80
3.80
3.79
3.78
3.71
3.69
2.24
0.69
1.95
2.00
2.11
0.97
1.05
0.98
1.00
1.04
1.05
0.97
1.06
1.08
3.04
f1 (ppm)

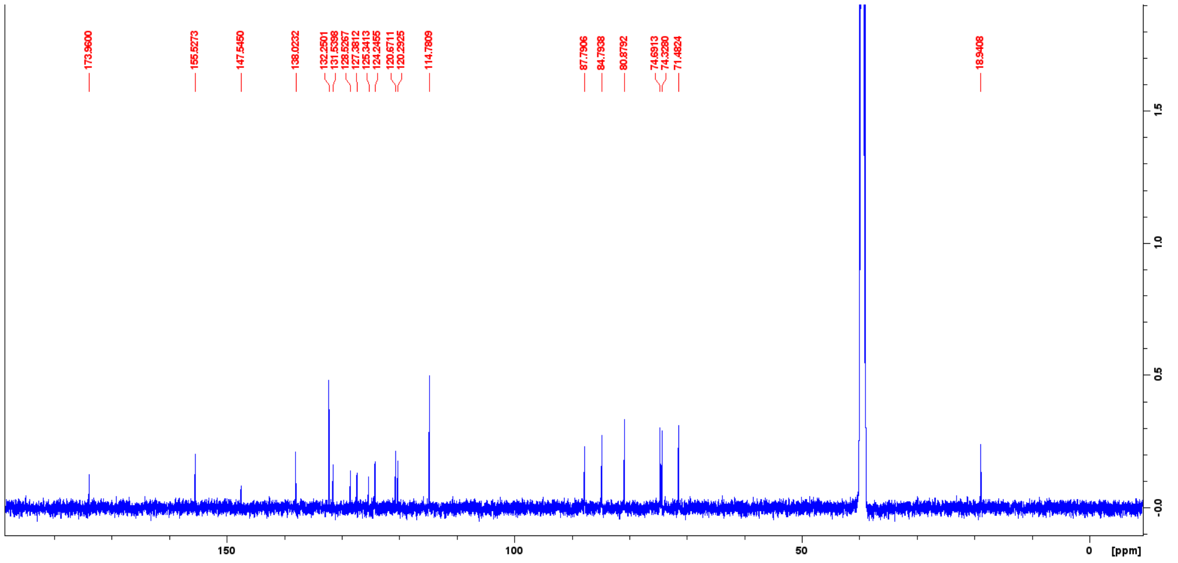
173.9600
155.5273
147.5450
138.0232
132.2501
131.5398
128.5267
127.3812
125.3413
124.2495
120.6711
120.2925
114.7809
87.7906
84.7938
80.8792
74.6913
74.3280
71.4824
18.9408
[ppm]

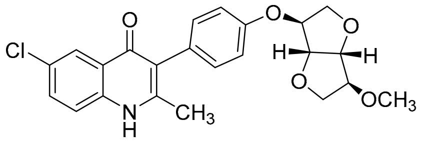
O
Cl
N
H
CH3
O
H
O
H
O
OCH3

16

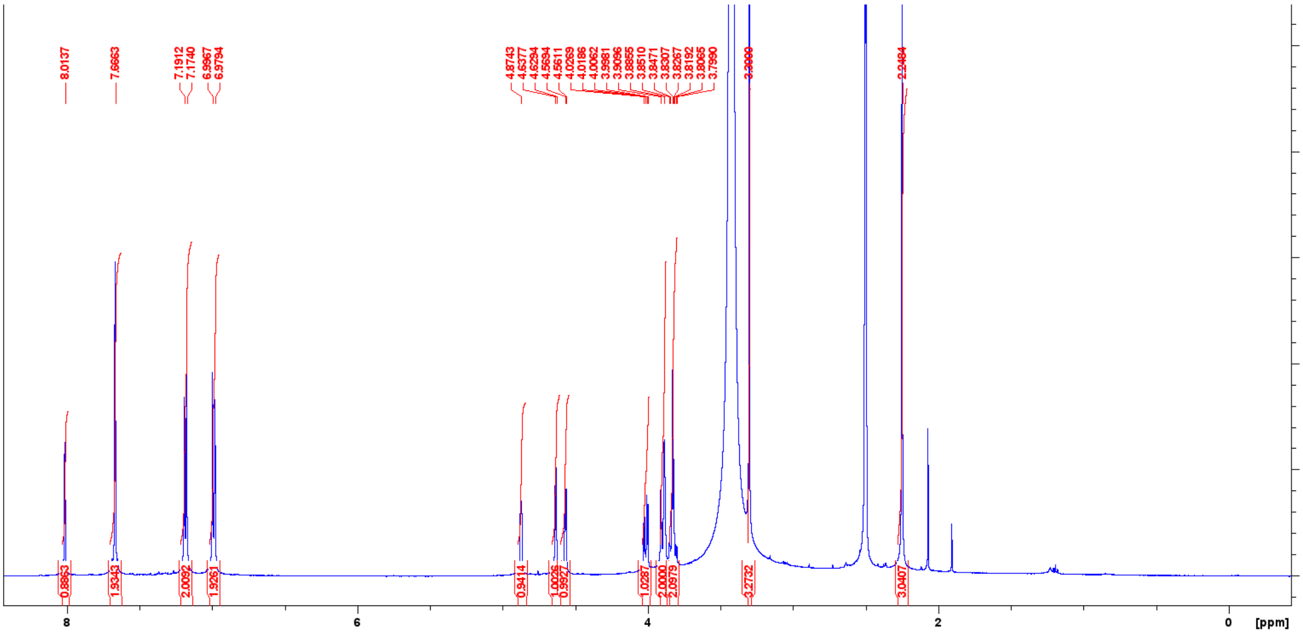
8.0137
7.6663
7.1912
7.1740
6.9967
6.9794
4.8743
4.6377
4.6294
4.5694
4.5611
4.0269
4.0186
4.0062
3.9981
3.9096
3.8855
3.8510
3.8471
3.8307
3.8267
3.8192
3.8065
3.7990
3.3000
2.2484
0.8863
1.9343
2.0092
1.9261
0.9414
1.0026
0.9927
1.0287
2.0000
2.0979
3.2732
3.0407
8
6
4
2
0
[ppm]
2.5
2.0
1.5
1.0
0.5
-0.0

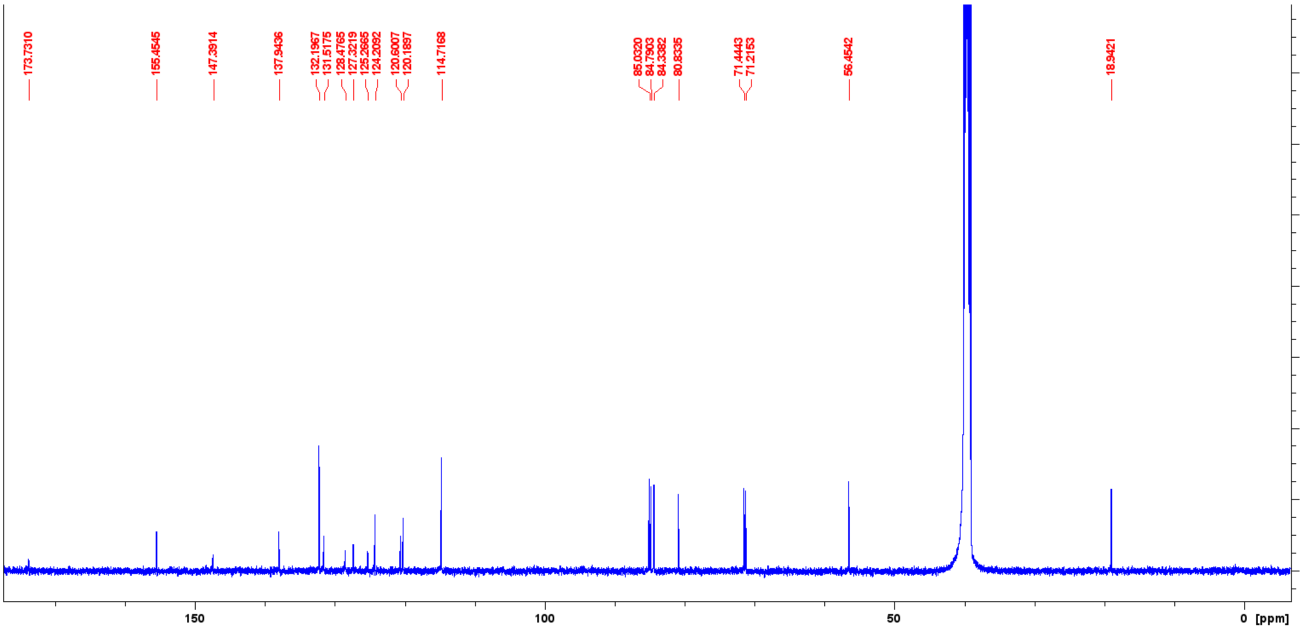
173.7310
155.4545
147.3914
137.9436
132.1967
131.5175
128.4765
127.3219
125.2665
124.2092
120.6007
120.1897
114.7168
85.0220
84.7903
84.3382
80.8335
71.4443
71.2153
56.4542
18.9421
150
100
50
0
[ppm]
1.4
1.2
1.0
0.8
0.6
0.4
0.2
-0.0

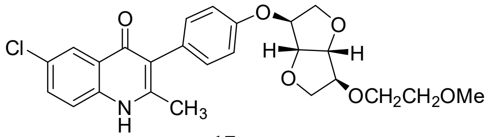
Cl
O
N
H
CH3
O
H
O
H
O
OCH2CH2OMe

17

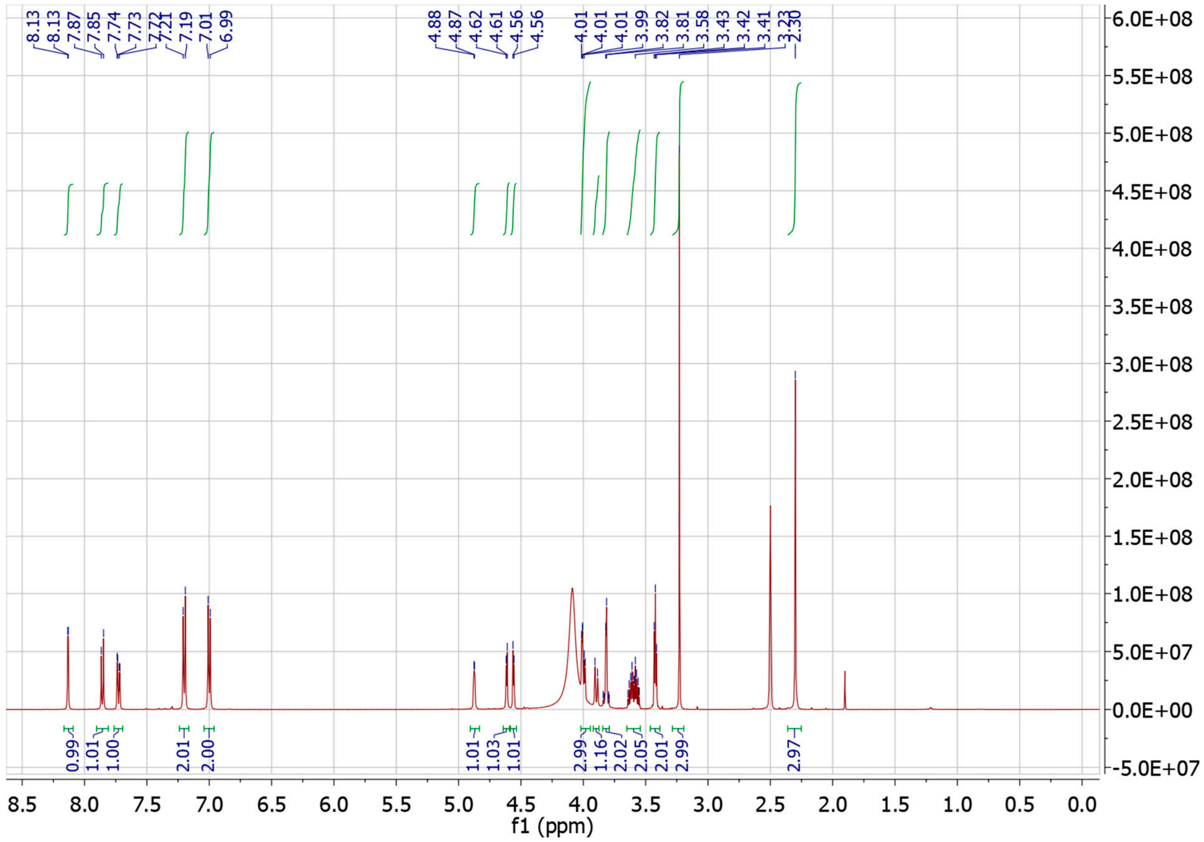
8.13
8.13
7.87
7.85
7.74
7.73
7.72
7.21
7.19
7.01
6.99
4.88
4.87
4.62
4.61
4.56
4.56
4.01
4.01
4.01
3.99
3.82
3.81
3.58
3.43
3.42
3.41
3.23
2.30
0.99
1.01
1.00
2.01
2.00
1.01
1.03
1.01
2.99
1.16
2.02
2.05
2.01
2.99
2.97
f1 (ppm)

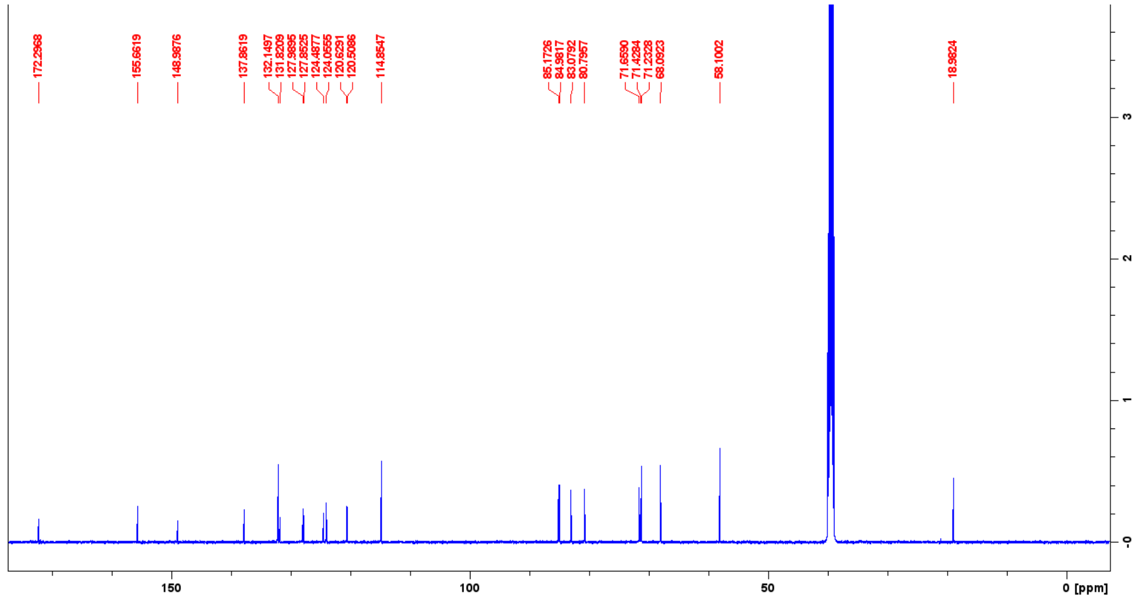
172.2968
155.6619
148.9876
137.8619
132.1497
131.8209
127.9895
127.8525
124.4877
124.0565
120.6291
120.5086
114.8547
85.1726
84.2617
83.0792
80.7957
71.6590
71.4284
71.2328
68.0923
58.1002
18.9824
[ppm]
