# Supplementary material for: Incorporation of an Isohexide Subunit into the Endochin-like Quinolone Scaffold
Source: Molecules. 2024 Jul 31;29(15):3615. doi: 10.3390/molecules29153615 (PMC11314205; doi:10.3390/molecules29153615)
Supplement: Supplementary file 1 [file molecules-29-03615-s001.zip › MSAnalayis_SpencerMol[71].pptx]

## Slide 1
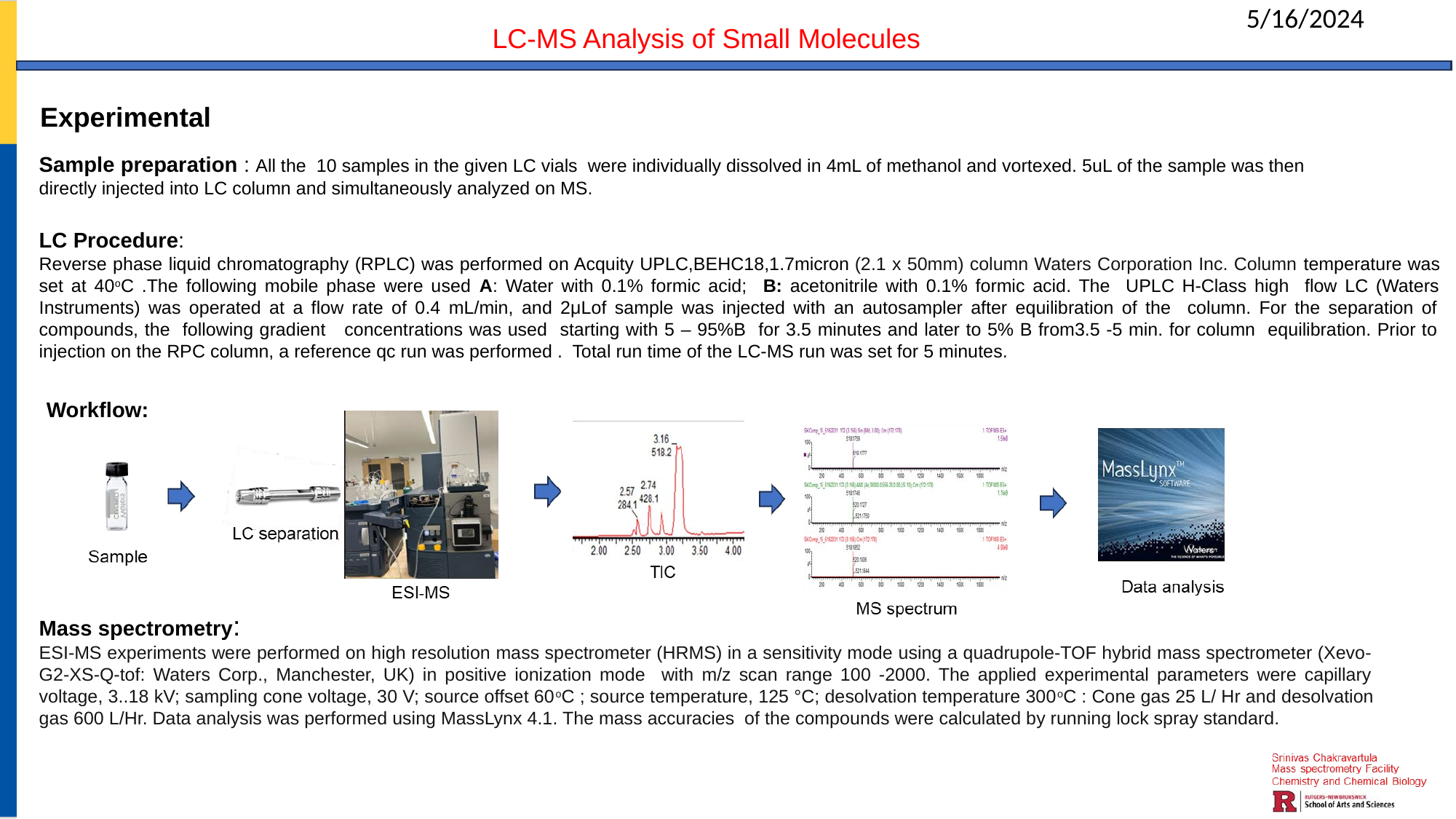

5/16/2024
LC-MS Analysis of Small Molecules
Experimental
Sample preparation : All the 10 samples in the given LC vials were individually dissolved in 4mL of methanol and vortexed. 5uL of the sample was then directly injected into LC column and simultaneously analyzed on MS.
LC Procedure:
Reverse phase liquid chromatography (RPLC) was performed on Acquity UPLC,BEHC18,1.7micron (2.1 x 50mm) column Waters Corporation Inc. Column temperature was set at 40oC .The following mobile phase were used A: Water with 0.1% formic acid; B: acetonitrile with 0.1% formic acid. The UPLC H-Class high flow LC (Waters Instruments) was operated at a flow rate of 0.4 mL/min, and 2μLof sample was injected with an autosampler after equilibration of the column. For the separation of compounds, the following gradient concentrations was used starting with 5 – 95%B for 3.5 minutes and later to 5% B from3.5 -5 min. for column equilibration. Prior to injection on the RPC column, a reference qc run was performed . Total run time of the LC-MS run was set for 5 minutes.
Workflow:
Mass spectrometry:
ESI-MS experiments were performed on high resolution mass spectrometer (HRMS) in a sensitivity mode using a quadrupole-TOF hybrid mass spectrometer (Xevo-G2-XS-Q-tof: Waters Corp., Manchester, UK) in positive ionization mode with m/z scan range 100 -2000. The applied experimental parameters were capillary voltage, 3..18 kV; sampling cone voltage, 30 V; source offset 60oC ; source temperature, 125 °C; desolvation temperature 300oC : Cone gas 25 L/ Hr and desolvation gas 600 L/Hr. Data analysis was performed using MassLynx 4.1. The mass accuracies of the compounds were calculated by running lock spray standard.

## Slide 2
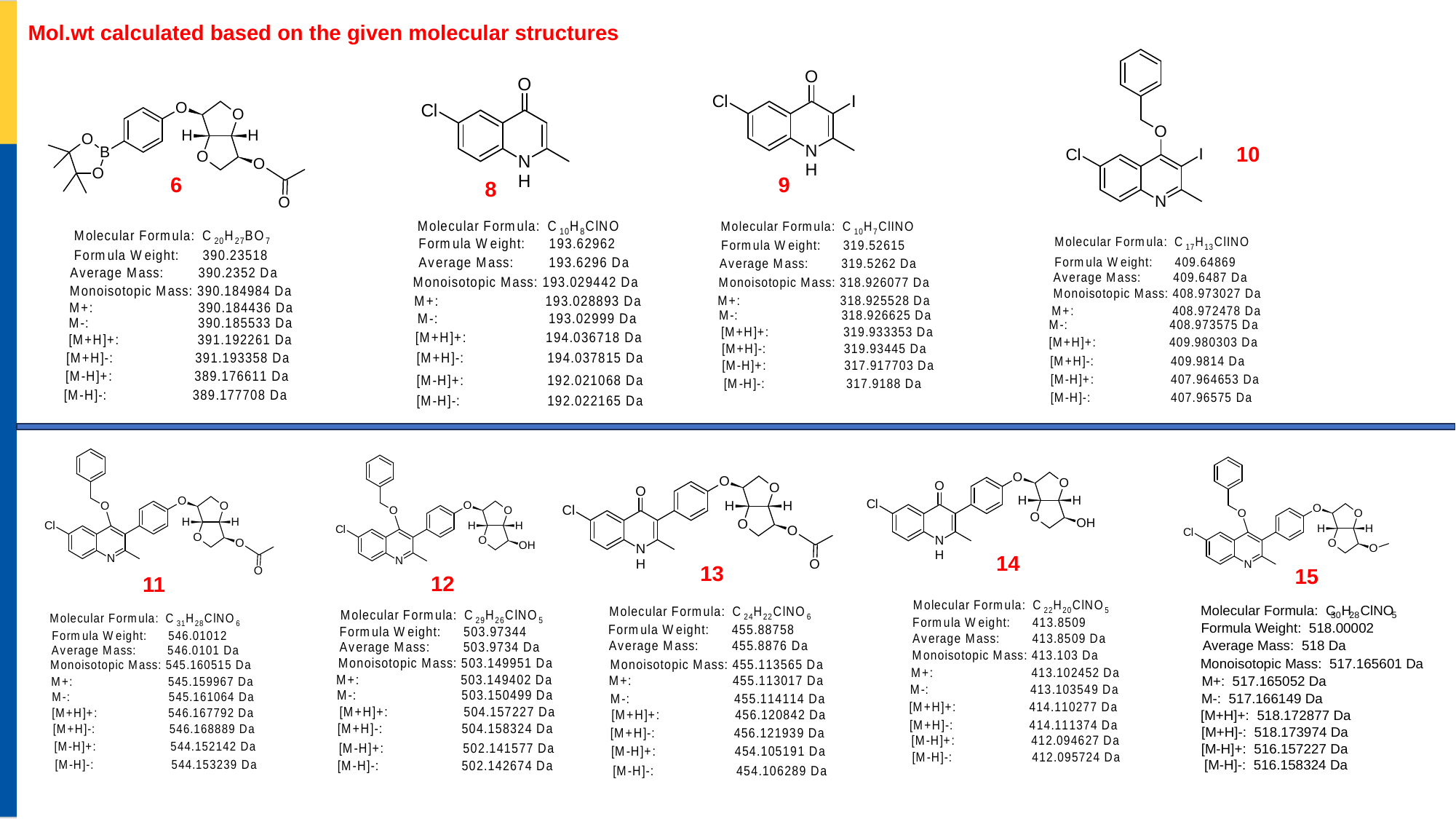

Mol.wt calculated based on the given molecular structures
10
9
6
8
14
13
15
12
11
Molecular Formula: C
H
ClNO
30
28
5
Formula Weight: 518.00002
Average Mass: 518 Da
Monoisotopic Mass: 517.165601 Da
M+: 517.165052 Da
M-: 517.166149 Da
[M+H]+: 518.172877 Da
[M+H]-: 518.173974 Da
[M-H]+: 516.157227 Da
[M-H]-: 516.158324 Da

## Slide 3
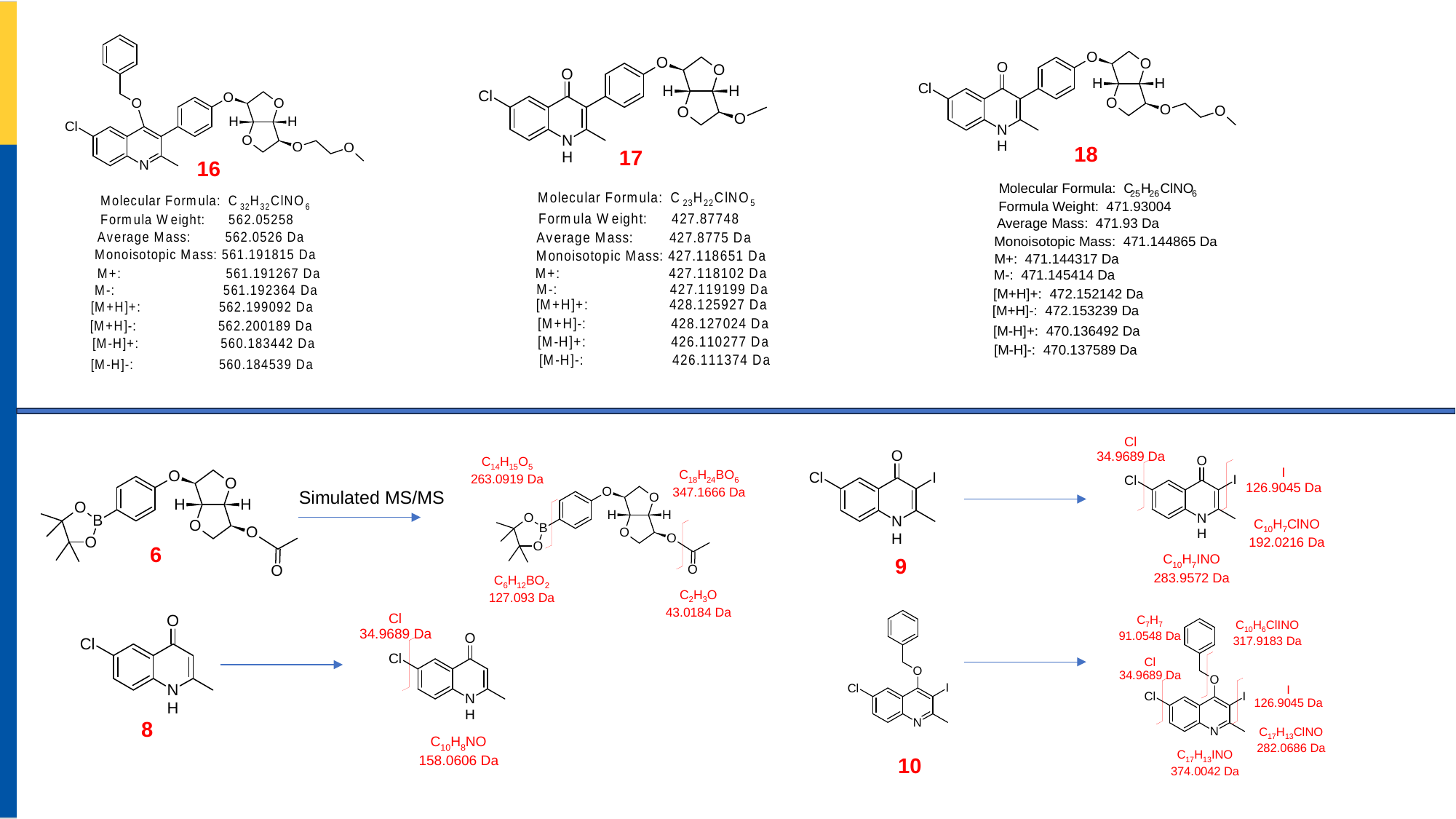

18
17
16
Molecular Formula: C
H
ClNO
25
26
6
Formula Weight: 471.93004
Average Mass: 471.93 Da
Monoisotopic Mass: 471.144865 Da
M+: 471.144317 Da
M-: 471.145414 Da
[M+H]+: 472.152142 Da
[M+H]-: 472.153239 Da
[M-H]+: 470.136492 Da
[M-H]-: 470.137589 Da
Simulated MS/MS
6
9
8
10

## Slide 4
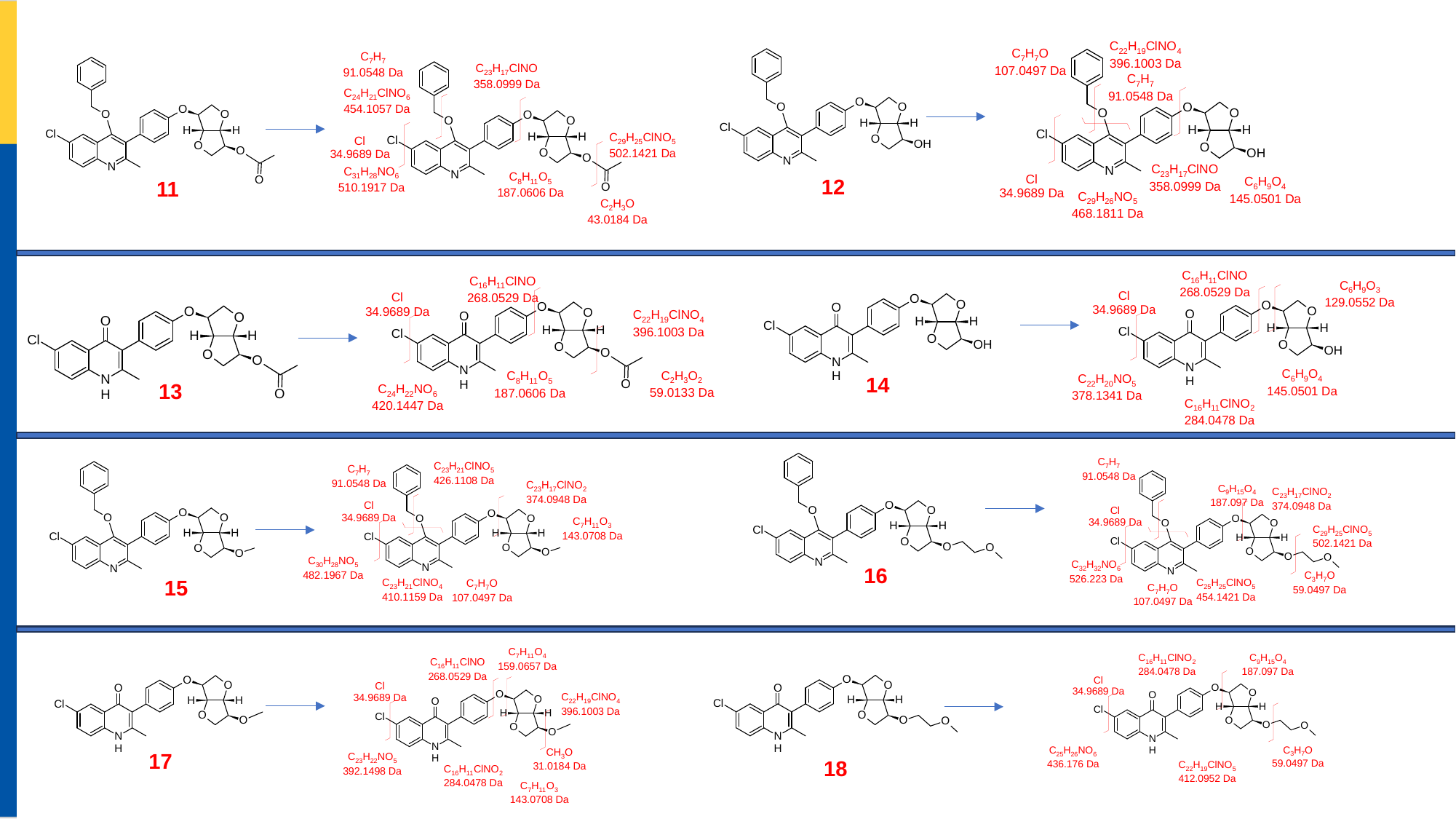

12
11
14
13
16
15
17
18

## Slide 5
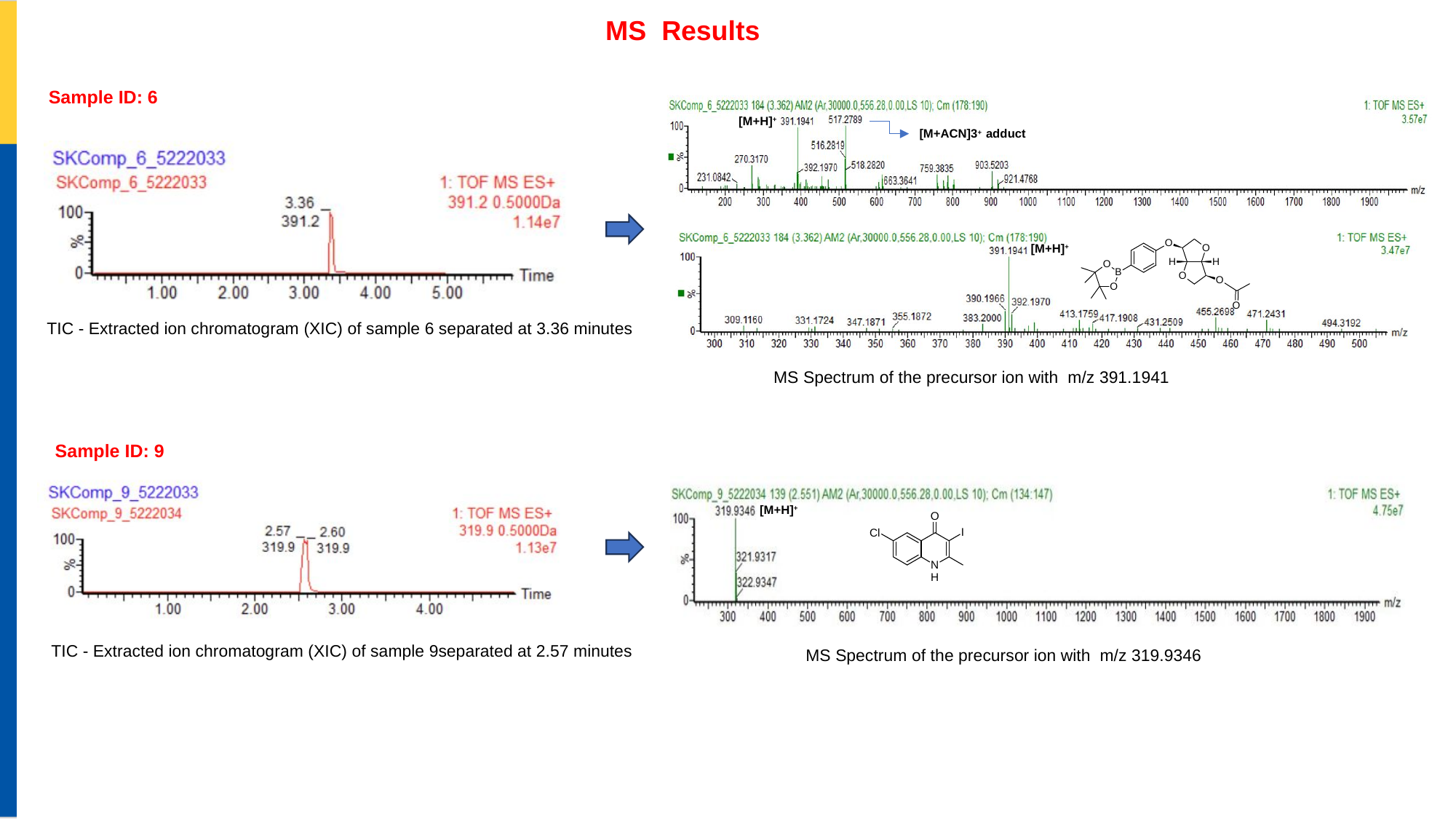

MS Results
Sample ID: 6
[M+H]+
[M+ACN]3+ adduct
[M+H]+
TIC - Extracted ion chromatogram (XIC) of sample 6 separated at 3.36 minutes
MS Spectrum of the precursor ion with m/z 391.1941
Sample ID: 9
[M+H]+
TIC - Extracted ion chromatogram (XIC) of sample 9separated at 2.57 minutes
MS Spectrum of the precursor ion with m/z 319.9346

## Slide 6
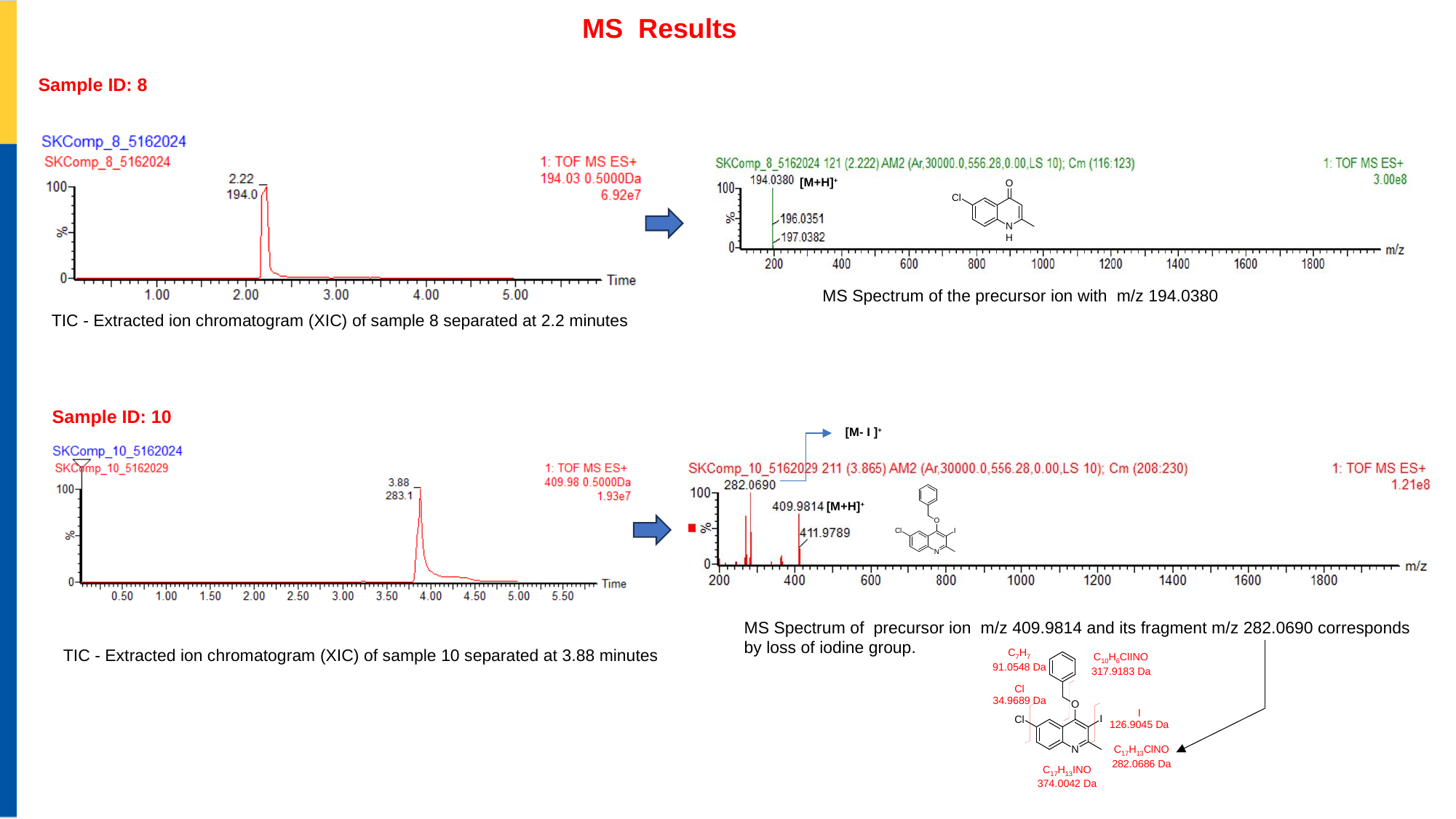

MS Results
Sample ID: 8
[M+H]+
MS Spectrum of the precursor ion with m/z 194.0380
TIC - Extracted ion chromatogram (XIC) of sample 8 separated at 2.2 minutes
Sample ID: 10
[M- I ]+
[M+H]+
MS Spectrum of precursor ion m/z 409.9814 and its fragment m/z 282.0690 corresponds
by loss of iodine group.
TIC - Extracted ion chromatogram (XIC) of sample 10 separated at 3.88 minutes

## Slide 7
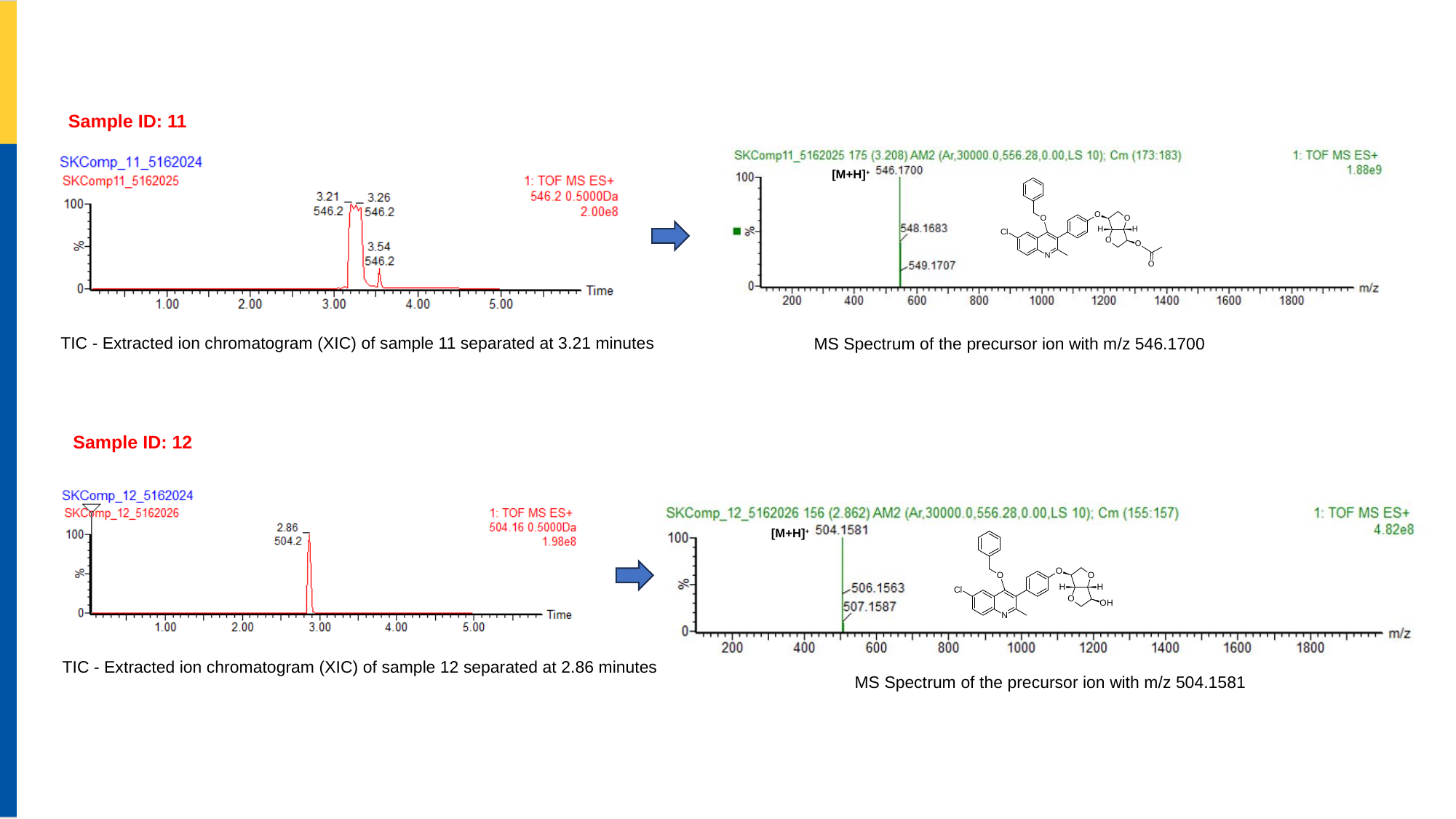

Sample ID: 11
[M+H]+
TIC - Extracted ion chromatogram (XIC) of sample 11 separated at 3.21 minutes
MS Spectrum of the precursor ion with m/z 546.1700
Sample ID: 12
[M+H]+
TIC - Extracted ion chromatogram (XIC) of sample 12 separated at 2.86 minutes
MS Spectrum of the precursor ion with m/z 504.1581

## Slide 8
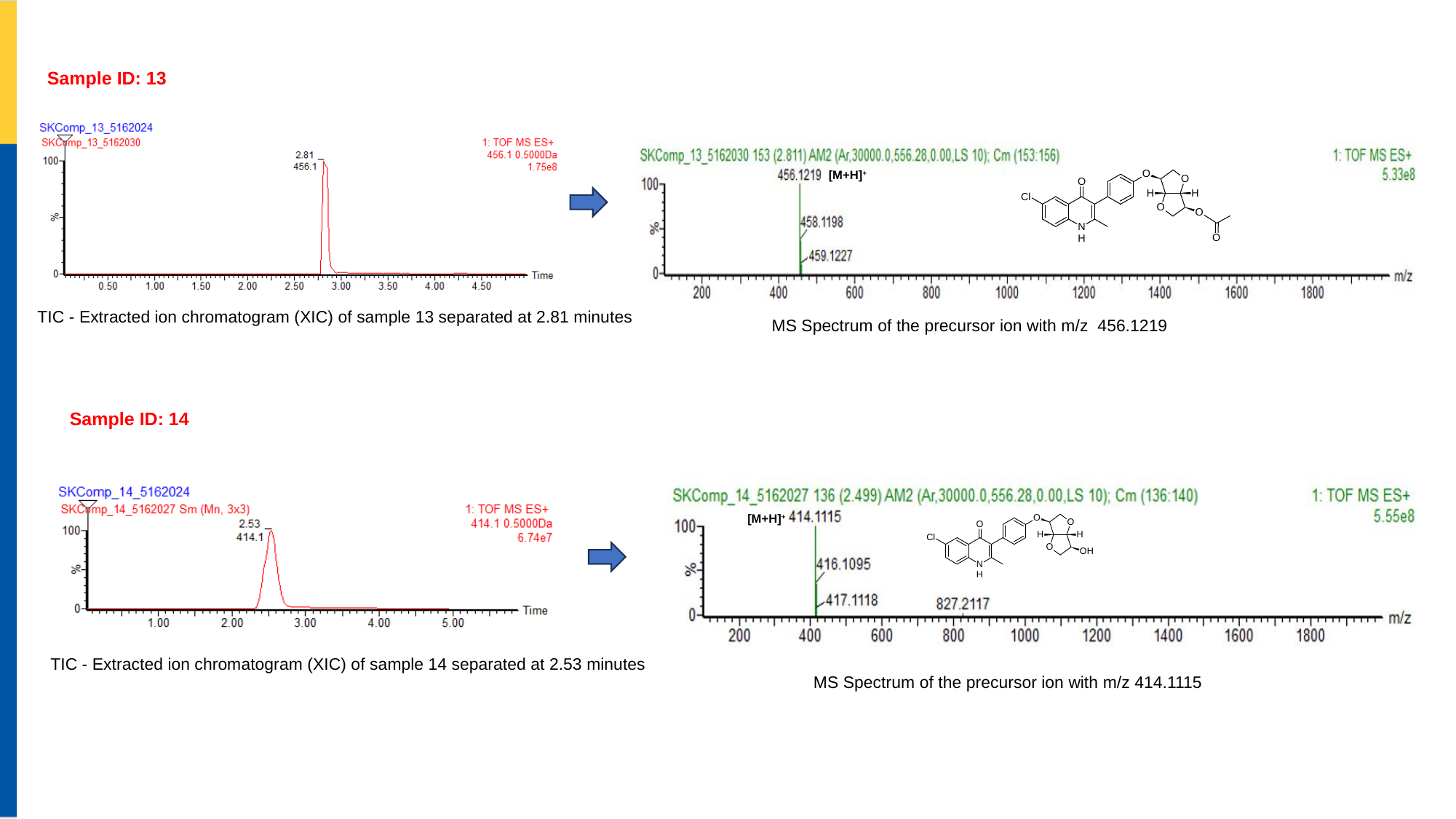

Sample ID: 13
[M+H]+
TIC - Extracted ion chromatogram (XIC) of sample 13 separated at 2.81 minutes
MS Spectrum of the precursor ion with m/z 456.1219
Sample ID: 14
[M+H]+
TIC - Extracted ion chromatogram (XIC) of sample 14 separated at 2.53 minutes
MS Spectrum of the precursor ion with m/z 414.1115

## Slide 9
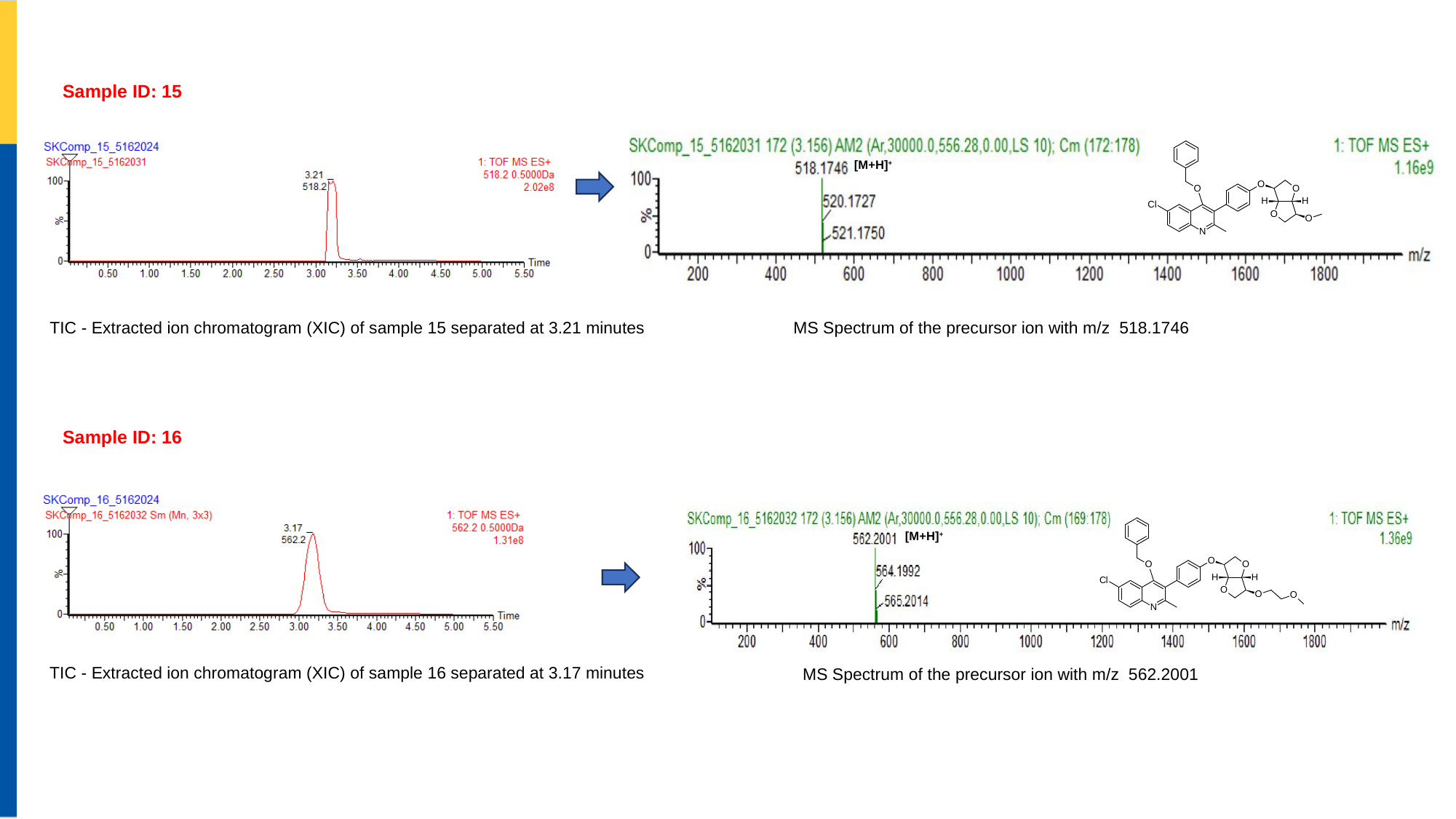

Sample ID: 15
[M+H]+
TIC - Extracted ion chromatogram (XIC) of sample 15 separated at 3.21 minutes
MS Spectrum of the precursor ion with m/z 518.1746
Sample ID: 16
[M+H]+
TIC - Extracted ion chromatogram (XIC) of sample 16 separated at 3.17 minutes
MS Spectrum of the precursor ion with m/z 562.2001

## Slide 10
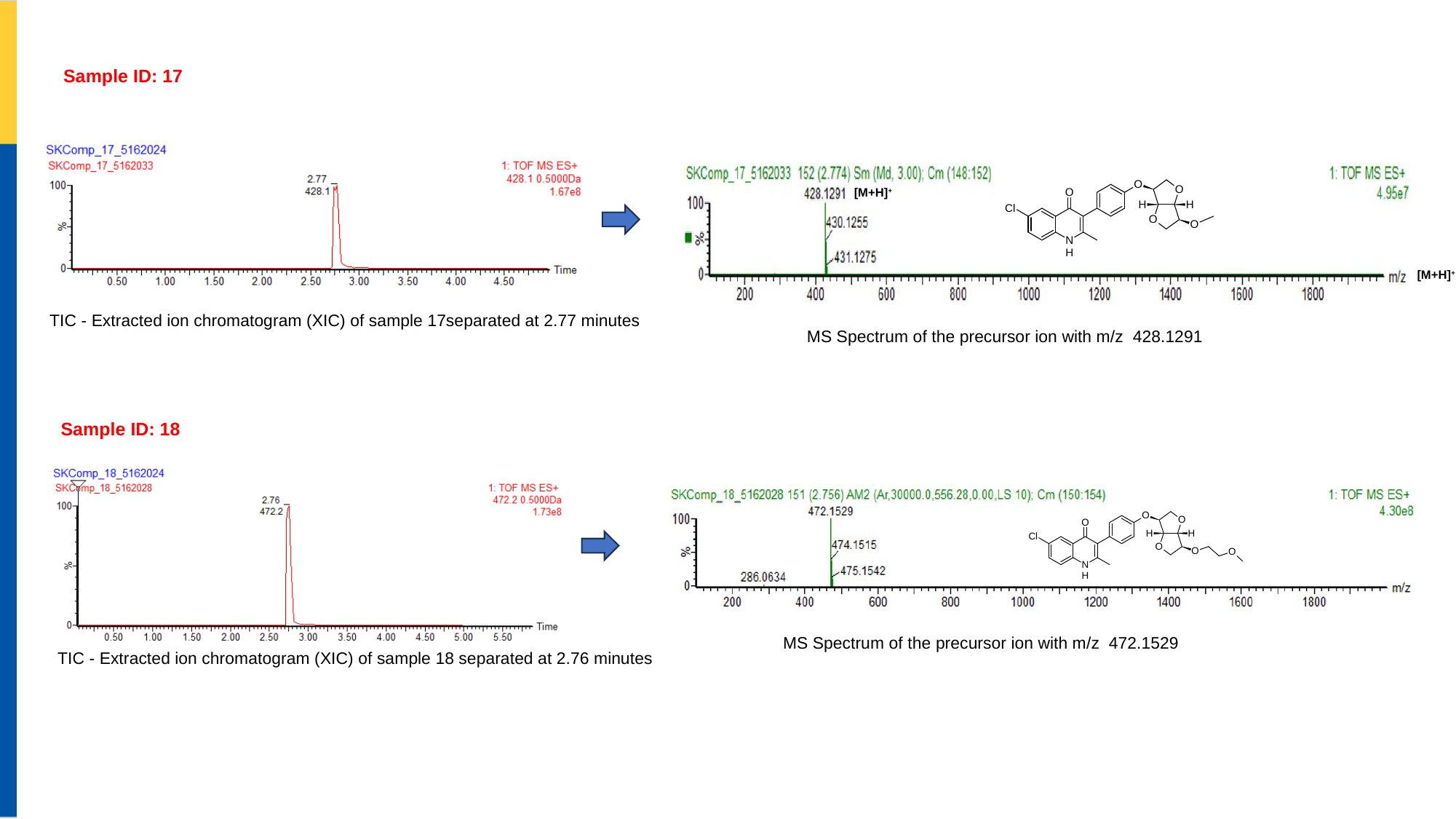

Sample ID: 17
[M+H]+
[M+H]+
TIC - Extracted ion chromatogram (XIC) of sample 17separated at 2.77 minutes
MS Spectrum of the precursor ion with m/z 428.1291
Sample ID: 18
MS Spectrum of the precursor ion with m/z 472.1529
TIC - Extracted ion chromatogram (XIC) of sample 18 separated at 2.76 minutes
