# Supplementary material for: Incorporation of an Isohexide Subunit into the Endochin-like Quinolone Scaffold
Source: Molecules. 2024 Jul 31;29(15):3615. doi: 10.3390/molecules29153615 (PMC11314205; doi:10.3390/molecules29153615)
Supplement: Supplementary file 1 [file molecules-29-03615-s001.zip › Zoltan ELQ Report_Solubility_MDS-20230316[41].pdf]

Thermodynamic Solubility Study in Phosphate Buffer 7.4

Compounds JS-13, JS-14, JS-17, JS-18

Prepared by	Zoltan Szekely PhD	***Zoltan Szekely*** ***02/24/2023*** [signature] ***(SIGNATURE & DATE)***

CONFIDENTIAL

Table of Contents

1 Introduction 3

1.1 Summary results 3

2 Method 3

2.1 Method description 4

3 Annex 5

3.1 Structures, molecular weights 5

3.2 Sample weights 6

3.3 Excel data files 7

CONFIDENTIAL

1 INTRODUCTION

The goal of this study is to determine the thermodynamic solubility values for compounds: JS-13, JS-14, JS-17 and JS-18 in phosphate buffer pH 7.4.

Results Summary:

Compound ID	Solubility (μM)	Solubility (mg/mL)
**JS-13**	Unstable in buffer	ND
**JS-14**	95	0.039
**JS-17**	22	0.009
**JS-18**	127	0.060

1.1 Summary results

JS-13 was hydrolyzed under equilibrating conditions.

JS-14 solubility: 94.56 μM (0.039 mg/mL)

JS-17 solubility: 21.96 μM (0.009 mg/mL)

JS-18 solubility: 126.97 μM (0.060 mg/mL)

2 METHOD

The scope of the thermodynamic (equilibrium) solubility experiment is to investigate the solubility of the compounds in a saturated solution. For this method, phosphate buffer is added to solid compound and mixed at room temperature for 24 h to ensure that equilibrium is achieved. To determine concentrations to generate calibration curves and for the equilibrated compounds (in buffer) a general LC-MS method was developed. The method includes a high-resolution separation by a UPLC unit as well as a high sensitivity ion detection by a mass spectrometer equipped with an electrospray ionization probe. For calibration curves the compounds were dissolved in DMSO and diluted by ACN. The supernatant of the equilibrated samples, after a 2-step centrifugation process, were also diluted by ACN to ensure full solubilization.

CONFIDENTIAL

2.1 Method description

The experiment was carried out as recommended by AstraZeneca's Mechanistic Biology & Profiling, Discovery Sciences, R&D group (AstraZeneca, Gothenburg, Sweden). The method was published in ASSAY and Drug Development Technologies; Title: "A Fully Integrated Assay Panel for Early Drug Metabolism and Pharmacokinetics Profiling" VOL. 18 NO. 4 MAY/JUNE 2020 p. 157-179.[1]

- The experiment was performed on December 3, 2022

- Instrument:

  A Shimadzu LC-40D XR HPLC system equipped with a PDA detector (Model: SPD-M40) and connected to an LCMS-2020 mass spectrometer was utilized for determining sample concentrations. For HPLC separation an XBridge C18 column (4.6x50 mm, 3.5 mm particle size) from Waters Inc. was used, the column oven temperature was set at 40°C. To determine the concentration of the compounds, eluent A containing 0.1% trifluoroacetic acid (TFA) in water, eluent B containing 0.1% TFA in acetonitrile and a linear gradient (5-100% B in 3 min) were utilized. Molecular ions (MH^+) were generated by combined ESI and APCI ionization methods and collected by a DUIS detector. The mass spectrometer was calibrated by standards provided by Shimadzu Scientific Instruments. Single ion monitoring (SIM) methods were used to maximize sensitivity for each compound as described in Table 1.

Compound ID	Primary Ion Monitored	Secondary Ion Monitored	Note
JS-13	465.15^+	414.15^+	414.15^+ is identical to MH^+ generated from JS-14, indicating hydrolysis
JS-14	414.15^+		
JS-17	428.20^+		
JS-18	472.20^+		

Table 1. SIM parameters

- To establish calibration curves, the weighted compounds were dissolved in 100 µL of dimethyl sulfoxide (DMSO) and diluted by 400 µL of acetonitrile (ACN). The stock solution was further diluted by ACN (5x, in 5 steps). For all LC-MS experiments 5 µL sample was injected. To perform equilibration studies, 500 µL phosphate buffer was added to each weighted compound. The results slurries were stirred at room temperature for 20 hr. After equilibration, the samples were transferred to

[1] DOI: 10.1089/adt.2020.970

CONFIDENTIAL

Eppendorf tubes and centrifuged for 30 min at 2,000 x *g*. 200 µL of the supernatant of each sample was transferred and centrifuged again for 30 min at 2,000 x *g*. Dilution and LC-MS analyses were performed as described for the calibration curves.

- Reagents

  ACN, DMSO and HPLC quality water were purchased from MilliporeSigma Inc.

  TFA (99.9% purity) was from Chem-Impex International Inc.

- The data was archived in the MD&S Laboratory electronic notebook (ChemCart, DeltaSoft Inc.) on page ZS-1-31.

- Data processing software used: Microsoft Office 365 – Excel.

3 ANNEX

3.1 Structures, molecular weights

Compound ID	Chemical Formula	Exact Mass	SMILE
**JS-13**	$C_{24}H_{22}ClNO_6$	455.1136	O=C1C(C2=CC=C(O[C@@H]3[C@](OC[C@@H]4OC(C)=O)([H])[C@]4([H])OC3)C=C2)=C(C)NC5=CC=C(Cl)C=C51
**JS-14**	$C_{22}H_{20}ClNO_5$	413.1030	O=C1C(C2=CC=C(O[C@@H]3[C@](OC[C@@H]4O)([H])[C@]4([H])OC3)C=C2)=C(C)NC5=CC=C(Cl)C=C51
**JS-17**	$C_{23}H_{22}ClNO_5$	427.1187	O=C1C(C2=CC=C(O[C@@H]3[C@](OC[C@@H]4OC)([H])[C@]4([H])OC3)C=C2)=C(C)NC5=CC=C(Cl)C=C51
**JS-18**	$C_{25}H_{26}ClNO_6$	471.1449	O=C1C(C2=CC=C(O[C@@H]3[C@](OC[C@@H]4OCCOC)([H])[C@]4([H])OC3)C=C2)=C(C)NC5=CC=C(Cl)C=C51

Table 2. Compound IDs, chemical formulas, exact (monoisotopic) molecular weights, SMILE notations

CONFIDENTIAL

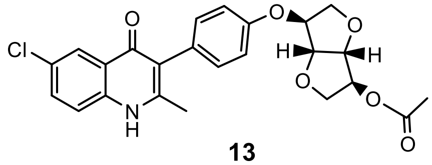

13

Chemical Formula: $C_{24}H_{22}ClNO_6$
Exact Mass: 455.1136
Molecular Weight: 455.8910

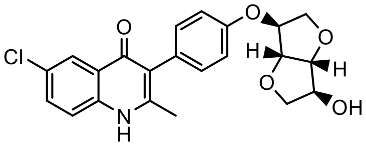

14

Chemical Formula: $C_{22}H_{20}ClNO_5$
Exact Mass: 413.1030
Molecular Weight: 413.8540

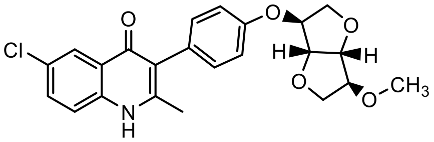

17

Chemical Formula: $C_{23}H_{22}ClNO_5$
Exact Mass: 427.1187
Molecular Weight: 427.8810

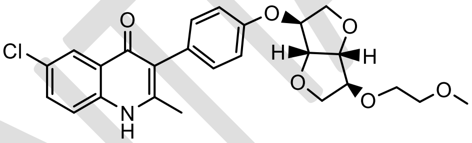

18

Chemical Formula: $C_{25}H_{26}ClNO_6$
Exact Mass: 471.1449
Molecular Weight: 471.9340

Figure 1. Chemical structures of the compounds analyzed

3.2 Sample weights

The following amounts were weighted out:

Compound ID	Amount for Calibration Curve mg	Amount for Equilibration mg
JS-13	0.76	1.61
JS-14	0.40	0.64
JS-17	1.29	1.48
JS-18	1.07	1.30

Table 3. Weights for the compounds analyzed.

CONFIDENTIAL

3.3 Excel data files

Sample	Concentration mM	Ion Integral
**1: 5x**	**0.3866**	5,192,652
**2: 25x**	**0.07732**	1,987,438
**3: 125x**	**0.015464**	1,028,215

Table 4. Calibration curve data for JS-14

Sample	Concentration mM	Ion Integral
**1: 5x**	**1.2059**	17,359,449
**2: 25x**	**0.24188**	9,535,464
**3: 125x**	**0.048238**	4,196,203
**4: 625x**	**0.0096475**	2,267,593
**5: 3125x**	**0.0019295**	1,376,329

Table 5. Calibration curve data for JS-17

Sample	Concentration mM	Ion Integral
**1: 5x**	**0.9069**	14,201,031
**2: 25x**	**0.18138**	6,242,241
**3: 125x**	**0.0363**	3,026,401
**4: 625x**	**0.00726**	1,498,205

Table 6. Calibration curve data for JS-18

Copy of JS14 SOL_ZS.xlsx

Copy of JS17 SOL_ZS.xlsx

Copy of JS18 SOL_ZS.xlsx

CONFIDENTIAL
